# Supplementary material for: Systematic discovery of novel phosphoinositide-binding effectors in Legionella reveals conserved ɑ-helical folds
Source: Microbiol Spectr. 2026 Jun 30;14(8):e03824-25. doi: 10.1128/spectrum.03824-25 (PMC13435680; doi:10.1128/spectrum.03824-25)
Supplement: Supplementary file 1 [file spectrum.03824-25-s0005.pdf]

Supplementary Information for

Systematic Discovery of Novel Phosphoinositide-Binding Effectors in *Legionella* Reveals Conserved α-Helical Folds

Abigail E. Bolt[1], Abby E. Richardson[1], Colleen M. Pike[1], Rebecca R. Noll[1], Yanbao Yu[2], Joshua P. Neunuebel[3], Sylvain Le Marchand[1,4], Karl R. Schmitz[1,2]*, and M. Ramona Neunuebel[1]*

[1]Department of Biological Sciences, University of Delaware, Newark, DE, USA 19716

[2]Department of Chemistry and Biochemistry, University of Delaware, Newark, DE, USA 19716

[3]Data Science Institute and Department of Psychological and Brain Sciences, University of Delaware, Newark, DE, USA 19716

[4]Delaware Biotechnology Institute, Bioimaging Center, University of Delaware, Newark, DE, USA 19713

*Co-corresponding Authors: Karl Schmitz[1,2] and M. Ramona Neunuebel[1]

Email: neunr@udel.edu

This PDF file includes:

- Figures S1 to S7
- Tables S1 to S3
- SI References

Other supporting materials for this manuscript include the following:

- Data S1: Pool maps and MS results
- Data S2: Mander's Overlap Coefficient analysis
- Data S3: FATCAT results *Legionella* effector analysis
- Data S4: FATCAT results *Coxiella* effector analysis

Supplementary Information: Figures

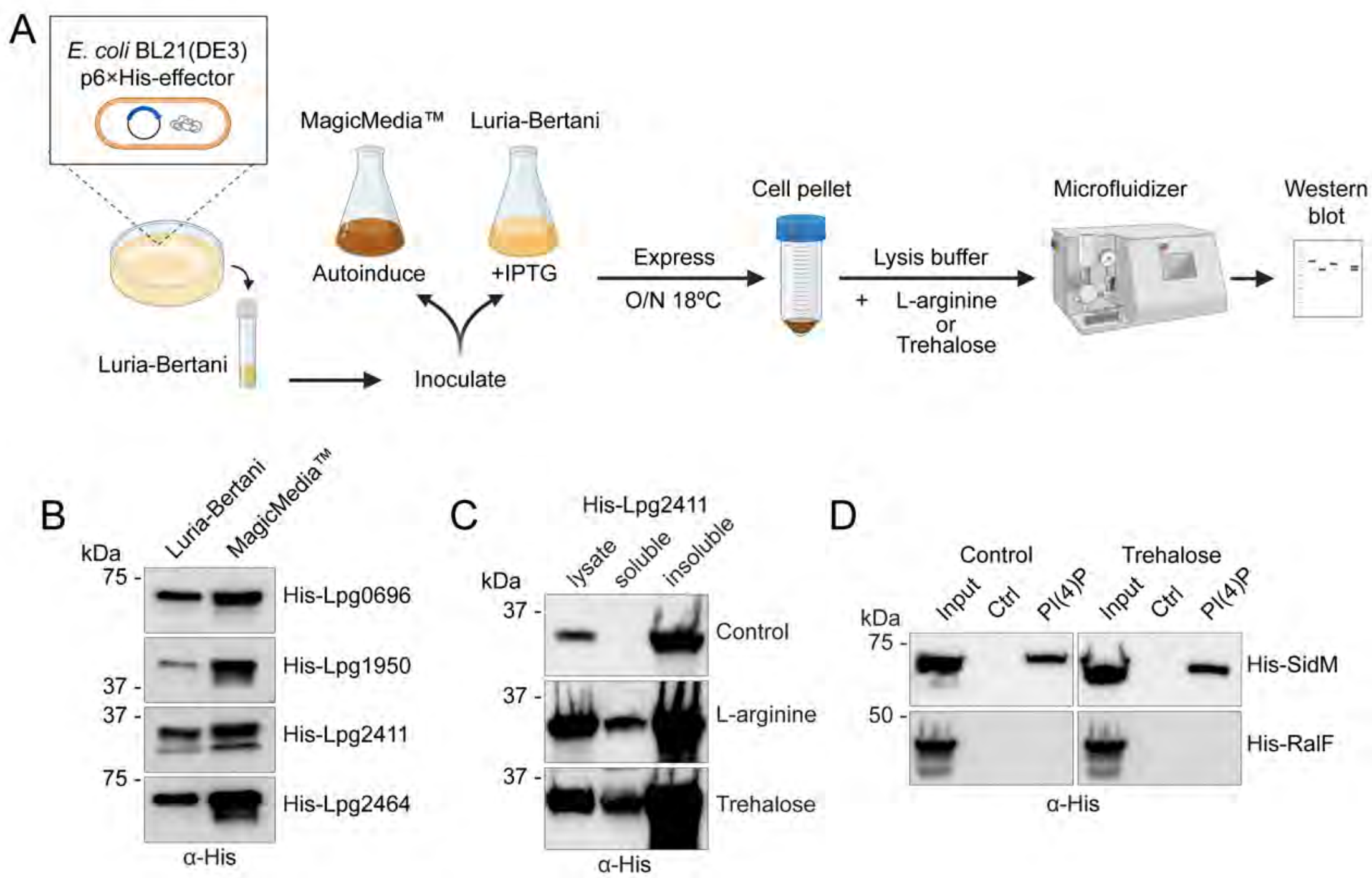

Fig. S1. Optimization of His-tagged effector protein expression and stability. (A) Workflow for effector expression and stability optimization. (B) Cultures of *E. coli* BL21(DE3) strains carrying plasmids encoding 6×His-tagged effectors were inoculated from overnight starter cultures in Luria-Bertani (LB) or MagicMedia™ and grown at 37°C. When an OD_{600} ~0.6 was reached, IPTG was added to the LB culture and both cultures were subsequently grown at 18°C overnight. Samples were normalized by volume, separated by SDS-PAGE, transferred to PVDF membrane, and probed with anti-His (mouse, 1:3,000). 6×His-Lpg0696/Lem3 ~65 kDa; 6×His-Lpg1950/RalF ~42 kDa; 6×His-Lpg2411/Lem24 ~33 kDa; 6×His-Lpg2464/SidM ~73 kDa (C) *E. coli* BL21(DE3) cells expressing 6×His-Lpg2411/Lem24 were collected after overnight growth at 18°C in LB. Cell pellets were resuspended in PBS-MM, PBS-MM + 300 mM L-arginine or PBS-MM + 750 mM trehalose and subsequently lysed with a microfluidizer. Whole cell lysate, soluble and insoluble fraction samples were separated by SDS-PAGE, transferred to PVDF membrane, and probed with anti-His (mouse, 1:3,000). (D) Proof of concept PIP-bead pulldown. *E. coli* BL21(DE3) lysate expressing 6×His-SidM or 6×His-RalF were incubated with agarose control beads or PI(4)P-coated beads. After incubation, beads were washed and elutions were separated via SDS-PAGE, transferred to PVDF membrane, and detected with anti-His (mouse, 1:3,000). 6×His-SidM ~ 73kDa; 6×His-RalF ~42 kDa.

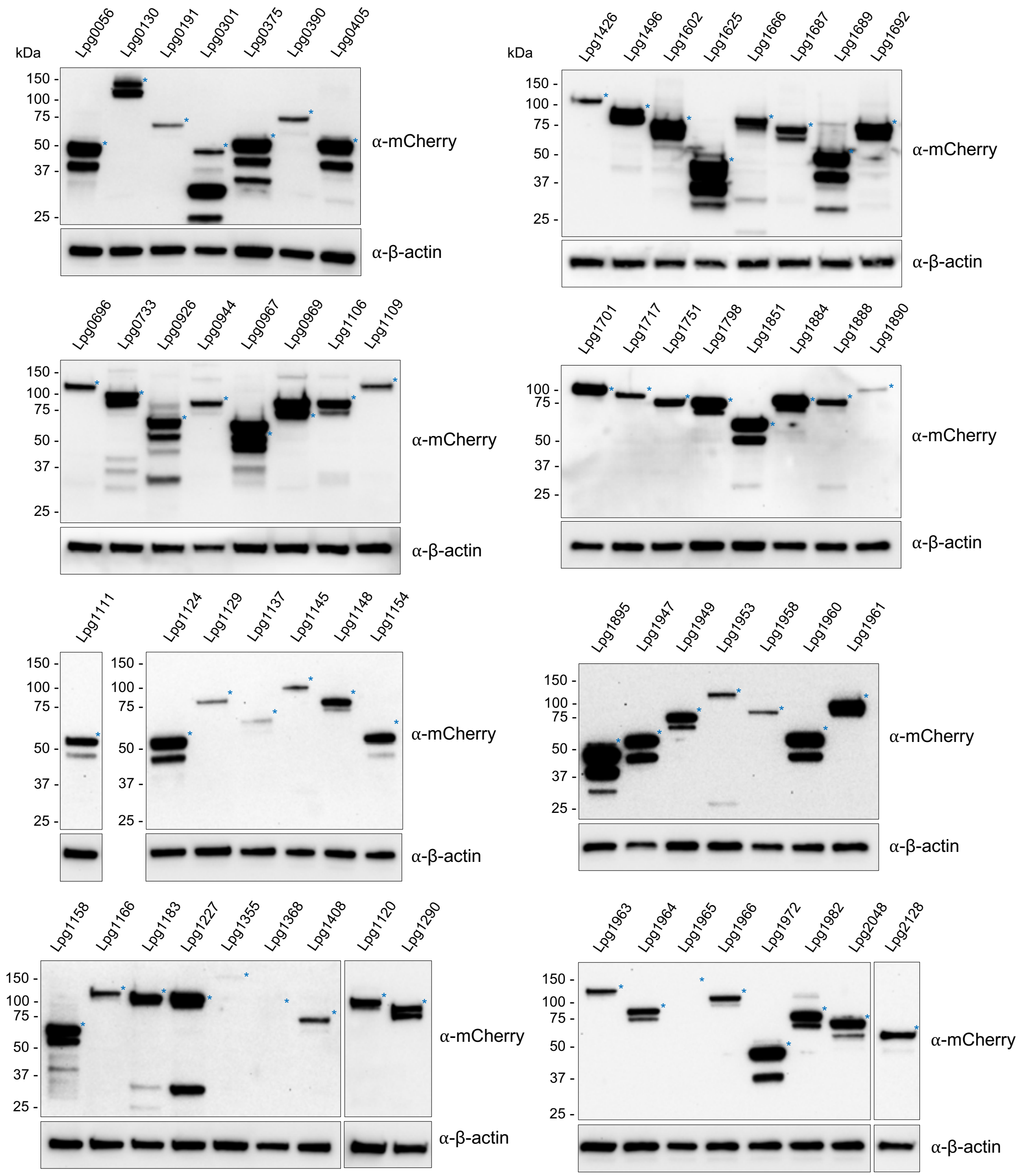
kDa
Lpg0056 Lpg0130 Lpg0191 Lpg0301 Lpg0375 Lpg0390 Lpg0405
150 - 100 - 75 - 50 - 37 - 25 -
α-mCherry
α-β-actin
kDa
Lpg1426 Lpg1496 Lpg1602 Lpg1625 Lpg1666 Lpg1687 Lpg1689 Lpg1692
150 - 100 - 75 - 50 - 37 - 25 -
α-mCherry
α-β-actin
Lpg0696 Lpg0733 Lpg0926 Lpg0944 Lpg0967 Lpg0969 Lpg1106 Lpg1109
150 - 100 - 75 - 50 - 37 - 25 -
α-mCherry
α-β-actin
Lpg1701 Lpg1717 Lpg1751 Lpg1798 Lpg1851 Lpg1884 Lpg1888 Lpg1890
100 - 75 - 50 - 37 - 25 -
α-mCherry
α-β-actin
Lpg1111
150 - 100 - 75 - 50 - 37 - 25 -
Lpg1124 Lpg1129 Lpg1137 Lpg1145 Lpg1148 Lpg1154
150 - 100 - 75 - 50 - 37 - 25 -
α-mCherry
α-β-actin
Lpg1895 Lpg1947 Lpg1949 Lpg1953 Lpg1958 Lpg1960 Lpg1961
150 - 100 - 75 - 50 - 37 - 25 -
α-mCherry
α-β-actin
Lpg1158 Lpg1166 Lpg1183 Lpg1227 Lpg1355 Lpg1368 Lpg1408 Lpg1120 Lpg1290
150 - 100 - 75 - 50 - 37 - 25 -
α-mCherry
α-β-actin
Lpg1963 Lpg1964 Lpg1965 Lpg1966 Lpg1972 Lpg1982 Lpg2048 Lpg2128
150 - 100 - 75 - 50 - 37 - 25 -
α-mCherry
α-β-actin

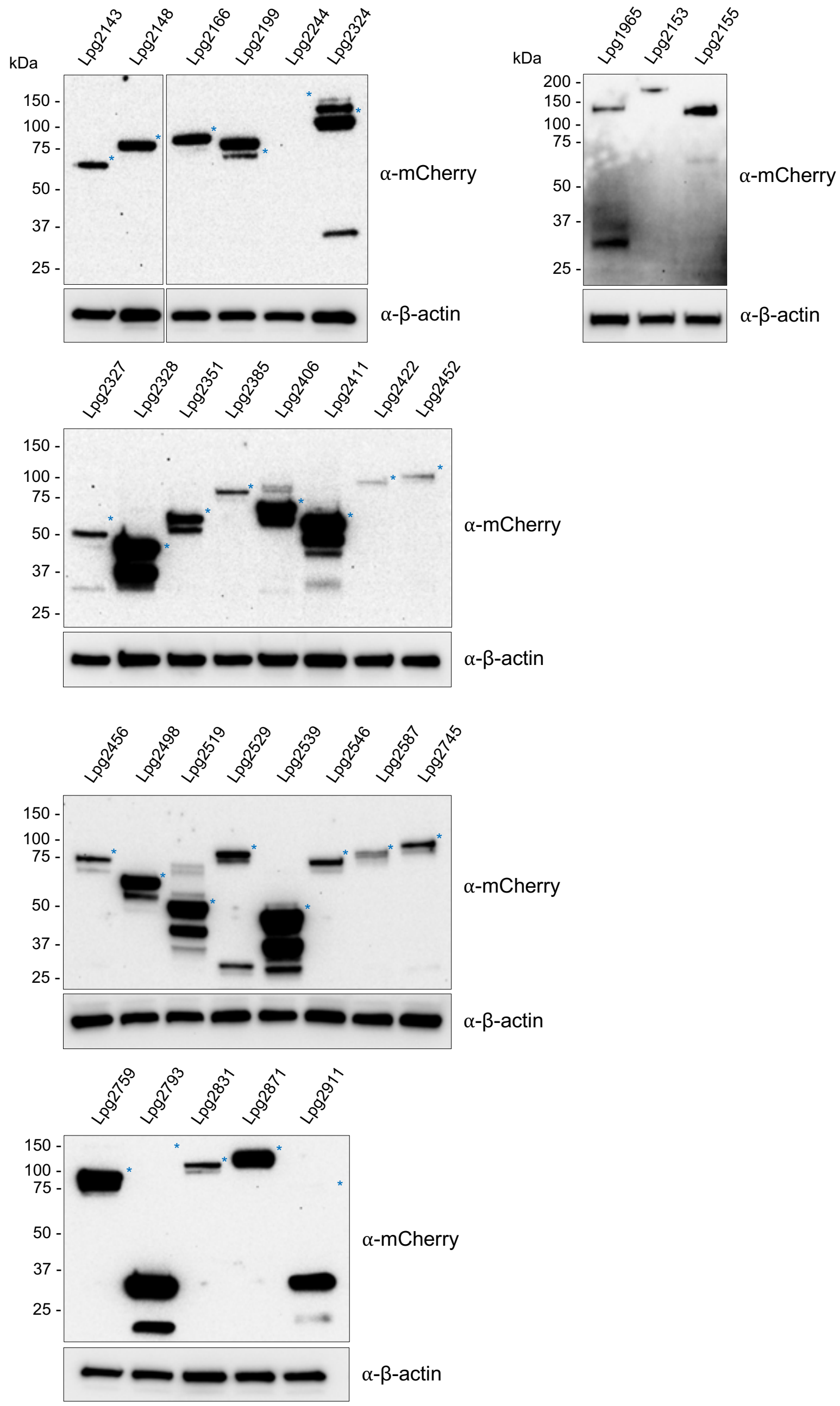

Fig. S2. mCherry-tagged effector fusion construct expression. HeLa cells transiently transfected with plasmids encoding mCherry-tagged effectors were lysed, lysate was separated via SDS-PAGE, transferred to a PVDF membrane, and probed with anti-mCherry (1:2,000) and anti-β-actin (1:2,000). Asterisks denote expected size of fusion construct.

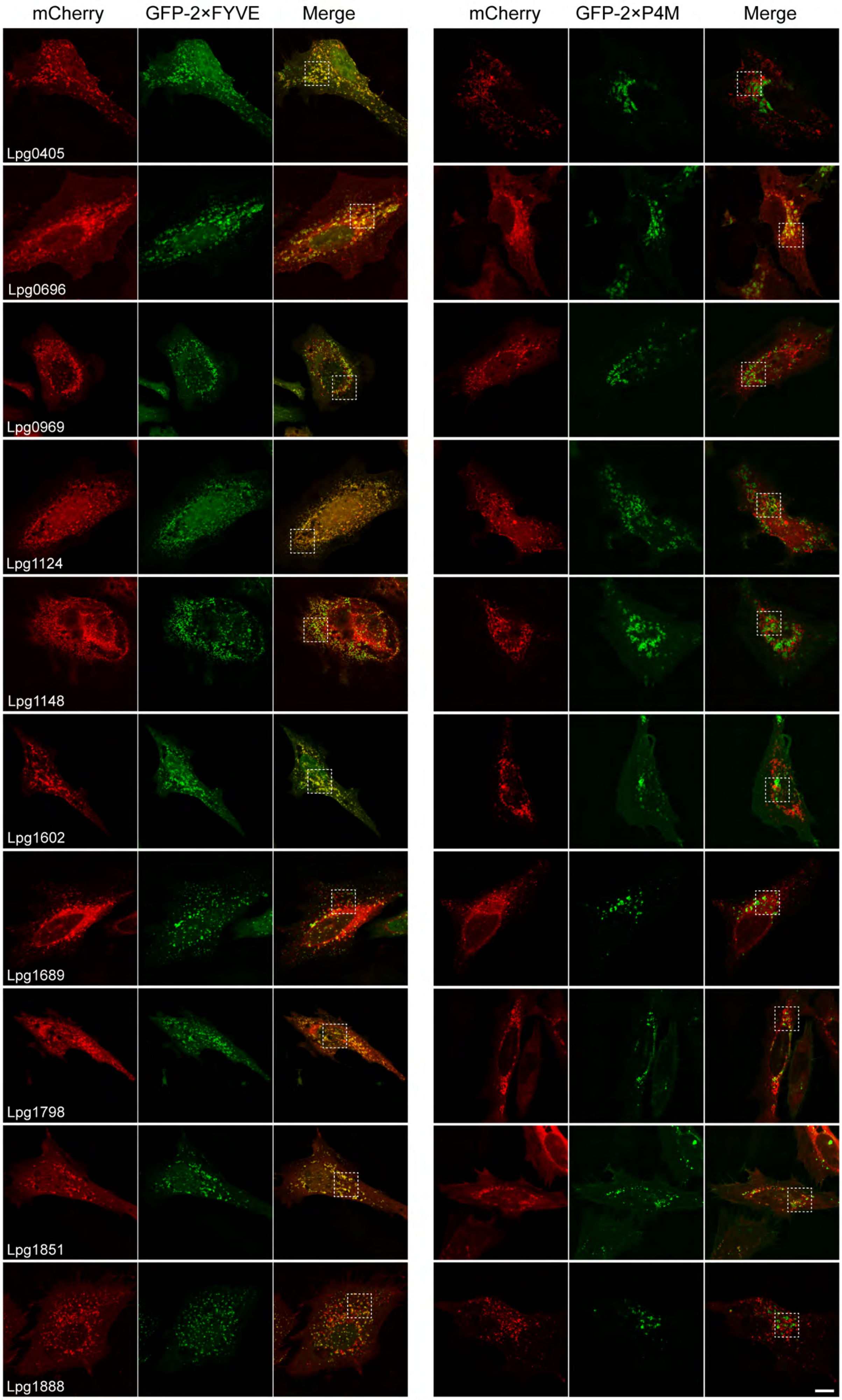
mCherry
GFP-2×FYVE
Merge
mCherry
GFP-2×P4M
Merge
Lpg0405
Lpg0696
Lpg0969
Lpg1124
Lpg1148
Lpg1602
Lpg1689
Lpg1798
Lpg1851
Lpg1888

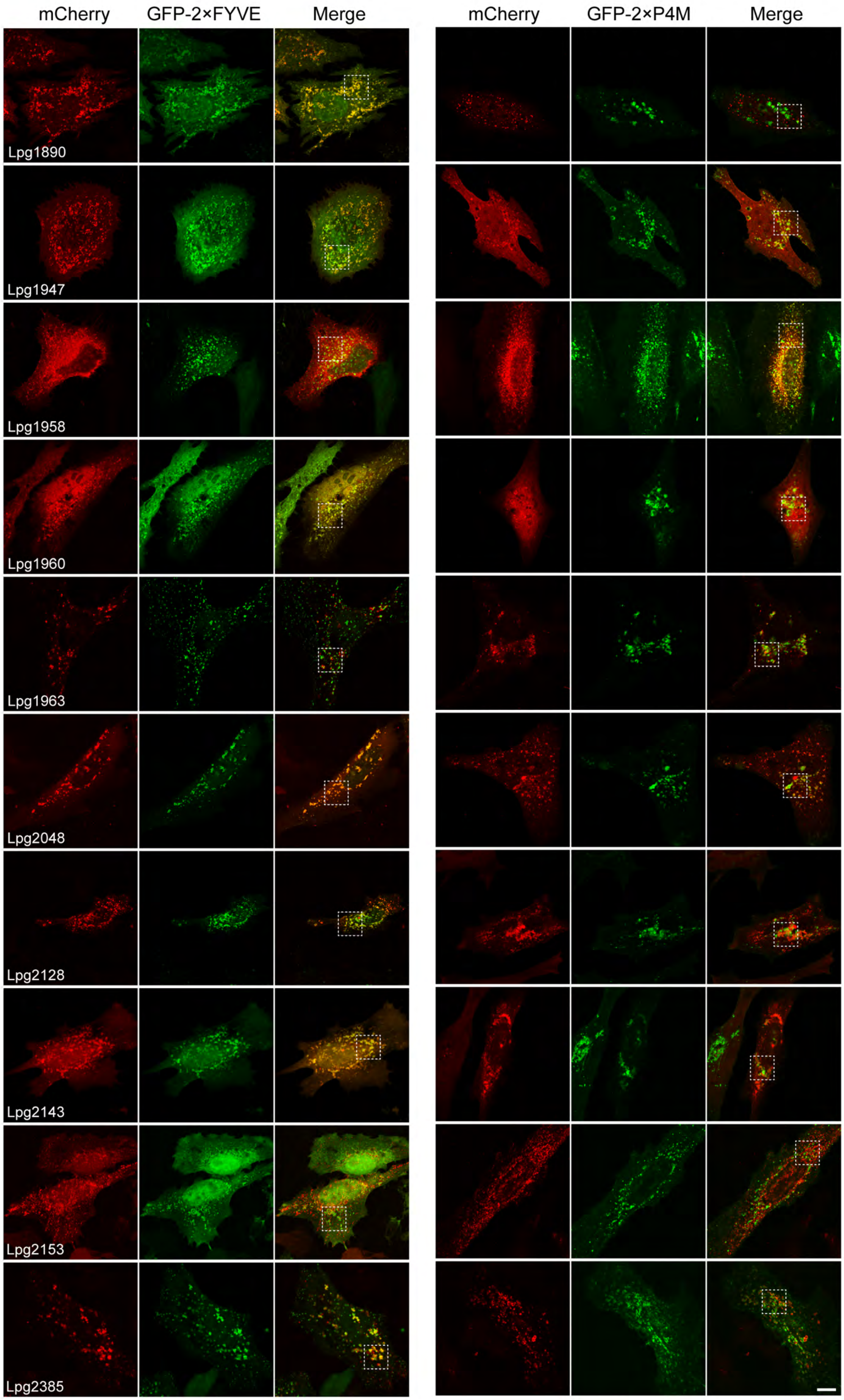
mCherry
GFP-2×FYVE
Merge
mCherry
GFP-2×P4M
Merge
Lpg1890
Lpg1947
Lpg1958
Lpg1960
Lpg1963
Lpg2048
Lpg2128
Lpg2143
Lpg2153
Lpg2385

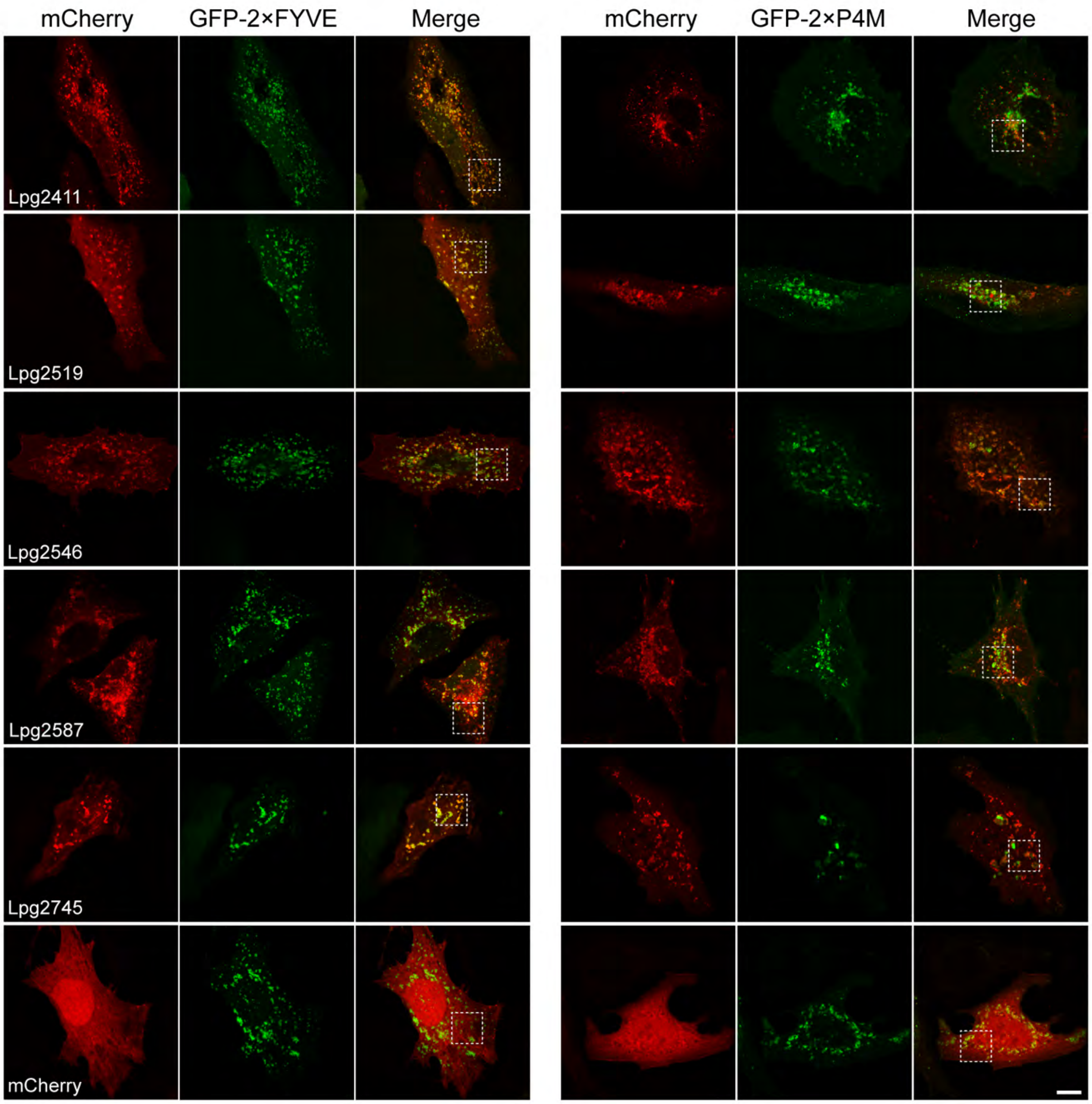

Fig. S3. Confocal images of mCherry-tagged effectors co-localizing with PIP-biosensor(s). HeLa cells were transiently co-transfected with plasmids encoding mCherry-tagged effectors and GFP-tagged PIP-biosensors, GFP-2×FYVE or GFP-2×P4M, were visualized with confocal microscopy. Regions of interest shown in Figure 2 are indicated by white box. Scale bars, 5μm.

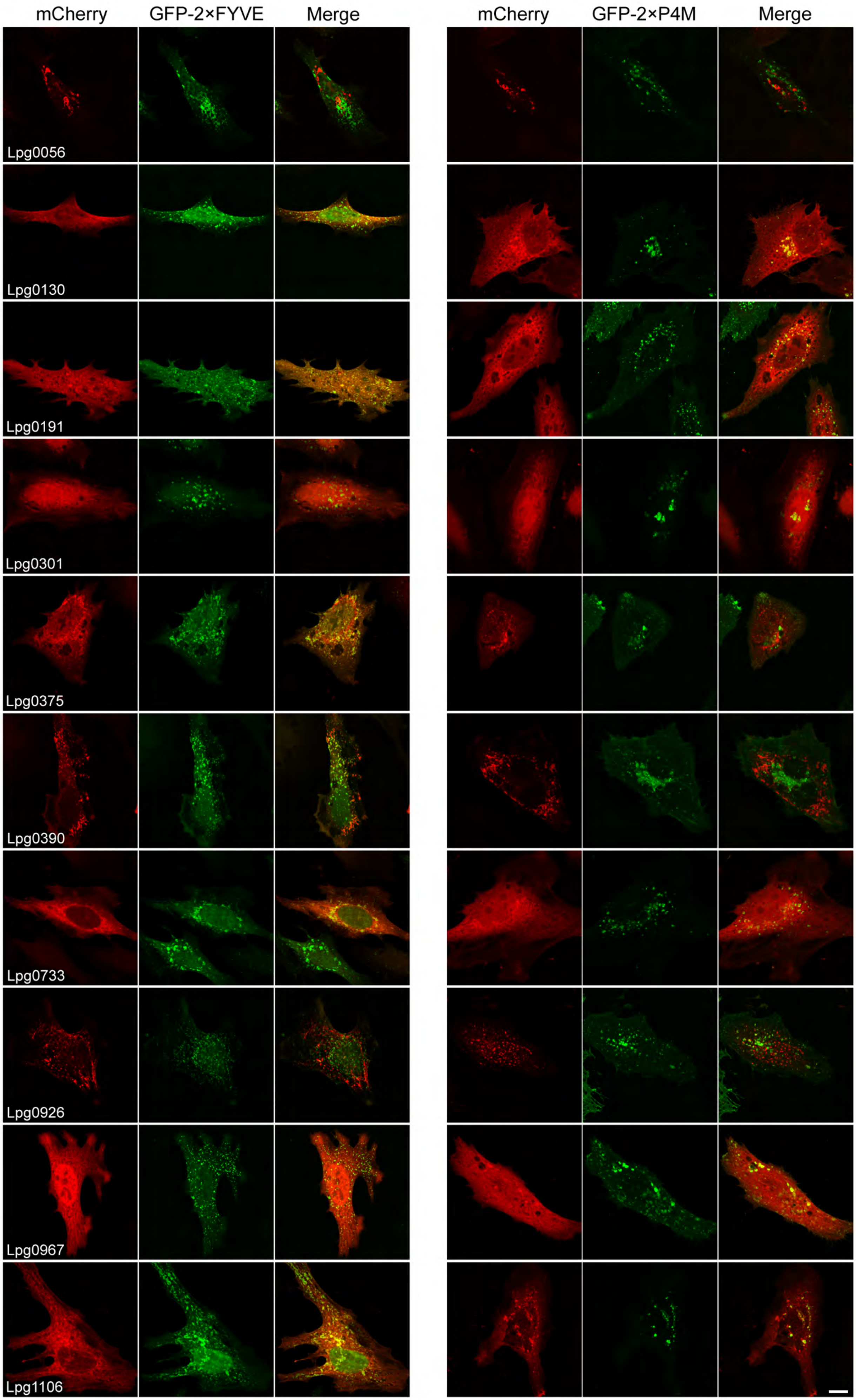

mCherry
GFP-2×FYVE
Merge
mCherry
GFP-2×P4M
Merge
Lpg0056
Lpg0130
Lpg0191
Lpg0301
Lpg0375
Lpg0390
Lpg0733
Lpg0926
Lpg0967
Lpg1106

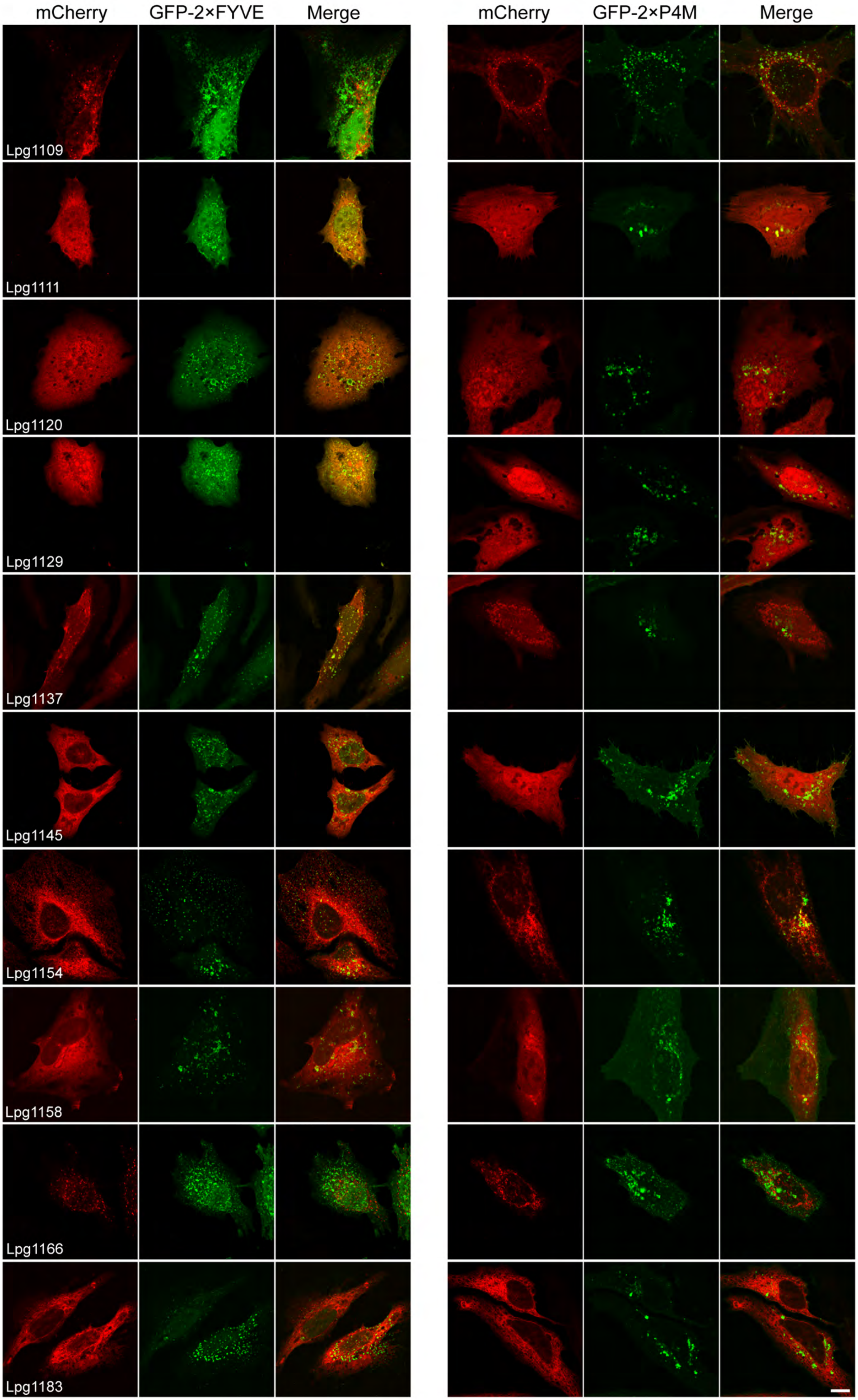
mCherry
GFP-2×FYVE
Merge
mCherry
GFP-2×P4M
Merge
Lpg1109
Lpg1111
Lpg1120
Lpg1129
Lpg1137
Lpg1145
Lpg1154
Lpg1158
Lpg1166
Lpg1183

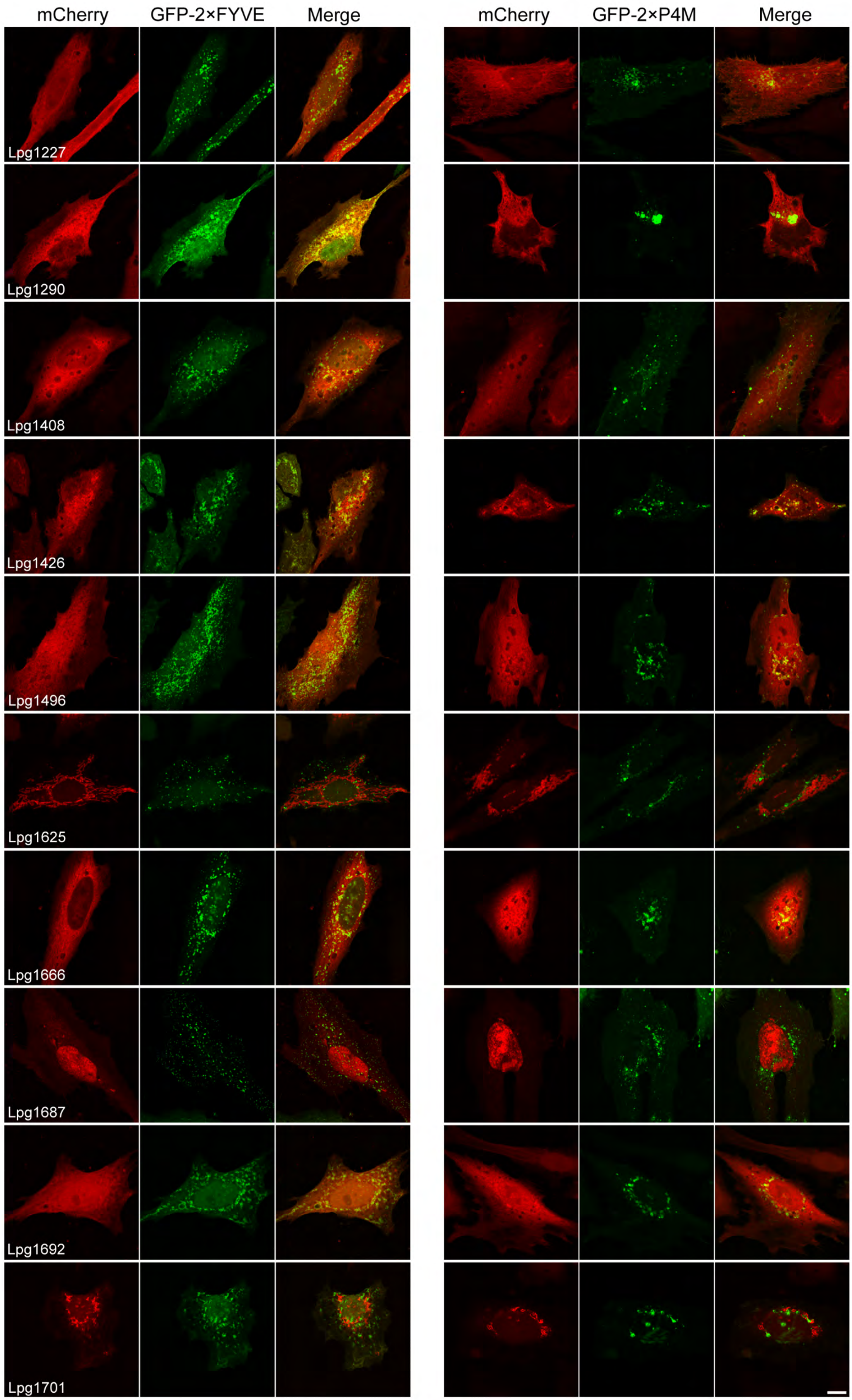
mCherry
GFP-2×FYVE
Merge
mCherry
GFP-2×P4M
Merge
Lpg1227
Lpg1290
Lpg1408
Lpg1426
Lpg1496
Lpg1625
Lpg1666
Lpg1687
Lpg1692
Lpg1701

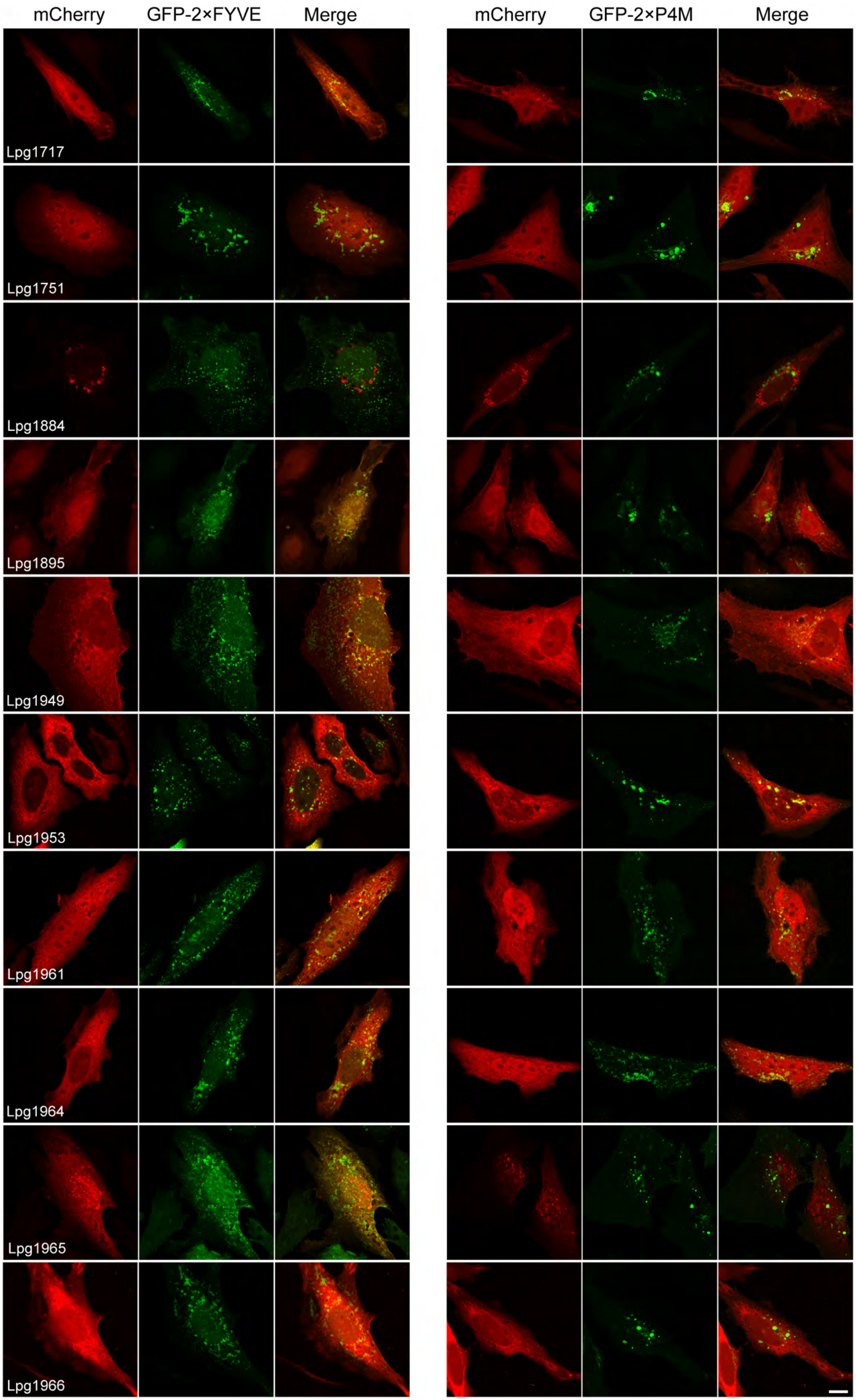
mCherry
GFP-2×FYVE
Merge
mCherry
GFP-2×P4M
Merge
Lpg1717
Lpg1751
Lpg1884
Lpg1895
Lpg1949
Lpg1953
Lpg1961
Lpg1964
Lpg1965
Lpg1966

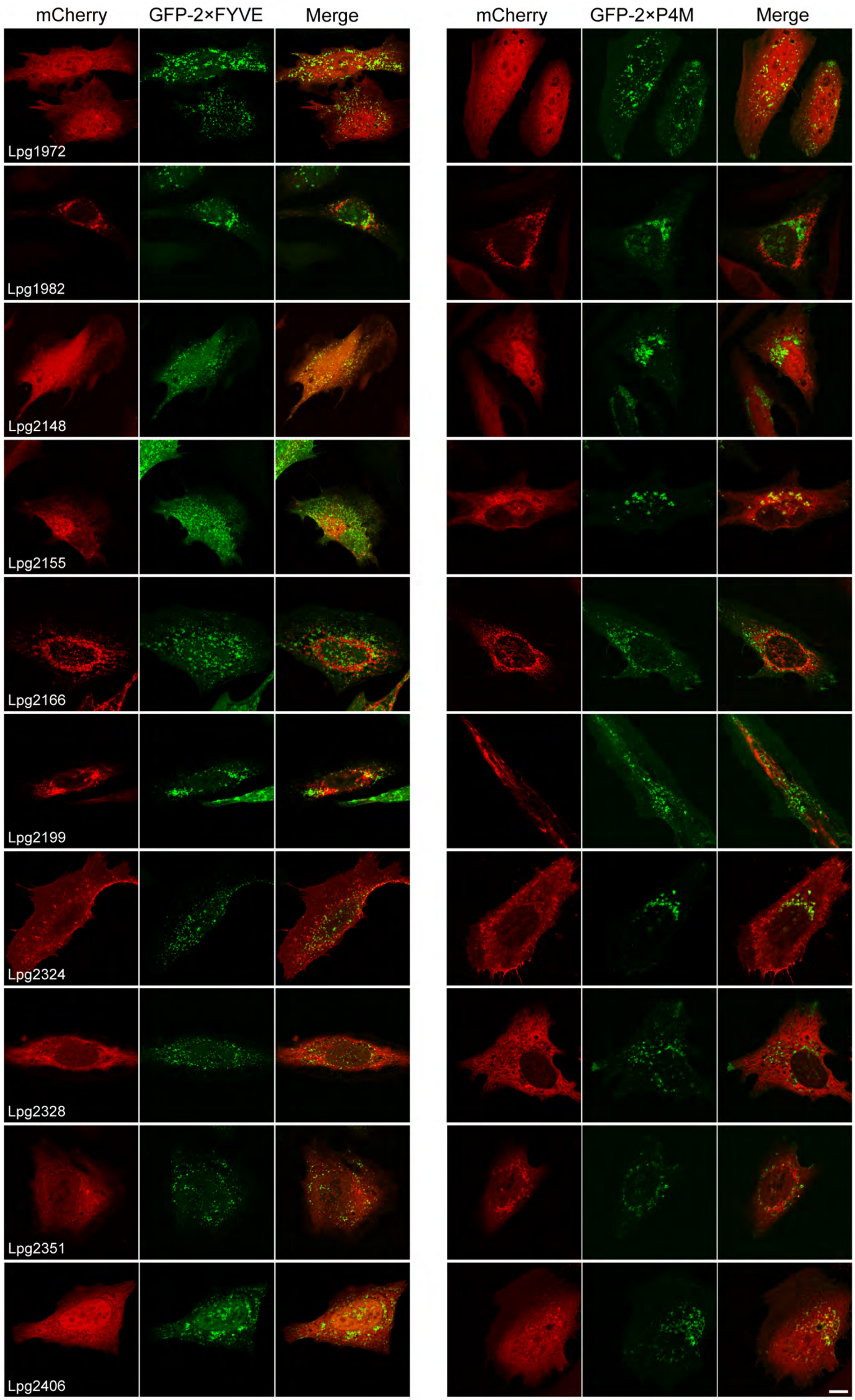
mCherry
GFP-2×FYVE
Merge
mCherry
GFP-2×P4M
Merge
Lpg1972
Lpg1982
Lpg2148
Lpg2155
Lpg2166
Lpg2199
Lpg2324
Lpg2328
Lpg2351
Lpg2406

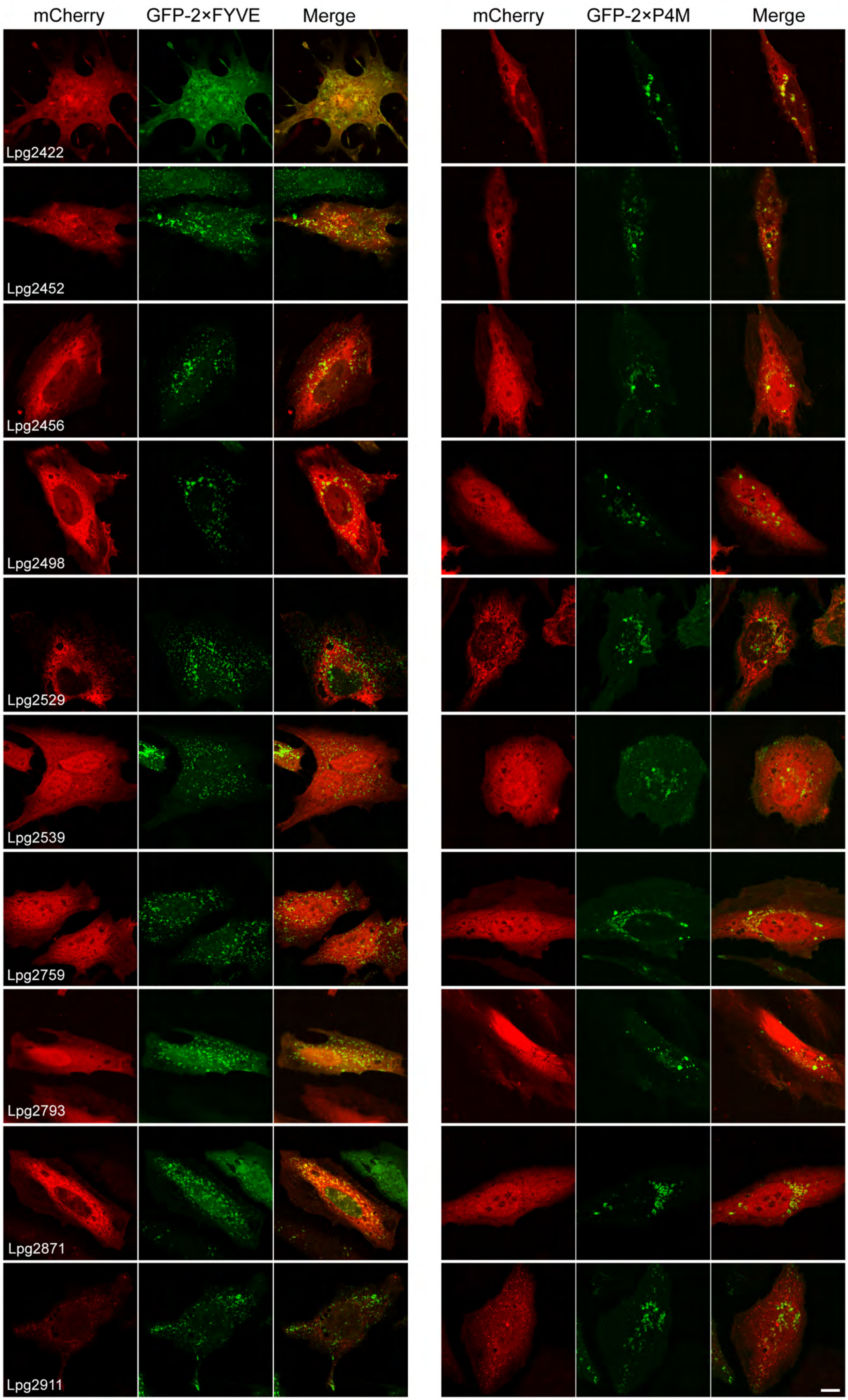
mCherry
GFP-2×FYVE
Merge
mCherry
GFP-2×P4M
Merge
Lpg2422
Lpg2452
Lpg2456
Lpg2498
Lpg2529
Lpg2539
Lpg2759
Lpg2793
Lpg2871
Lpg2911

Fig. S4. Confocal images of mCherry-tagged effectors that do not localize with PIP-biosensors. HeLa cells were transiently co-transfected with plasmids encoding mCherry-tagged effectors and GFP-tagged PIP-biosensors, GFP-2×FYVE or GFP-2×P4M, were visualized with confocal microscopy. Scale bars, 5μm.

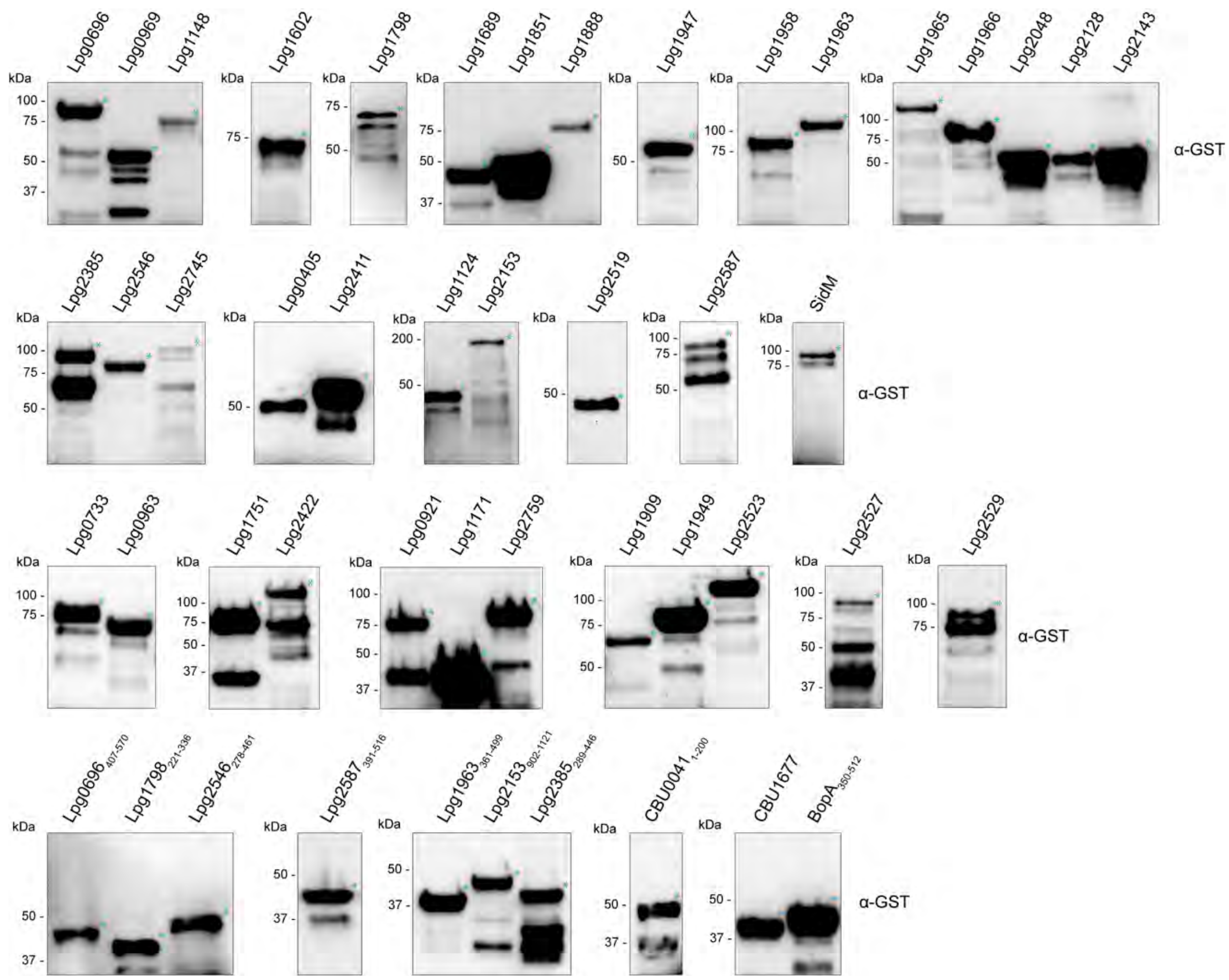

Fig. S5. Purified GST-fusion protein constructs. GST-fusions were expressed in *E. coli* BL21(DE3) strains, purified via affinity chromatography, separated via SDS-PAGE, transferred to a PVDF membrane, and probed with an anti-GST-HRP-conjugated antibody (1:3,000). Asterisks indicate the expected molecular weight of the full-length fusion proteins.

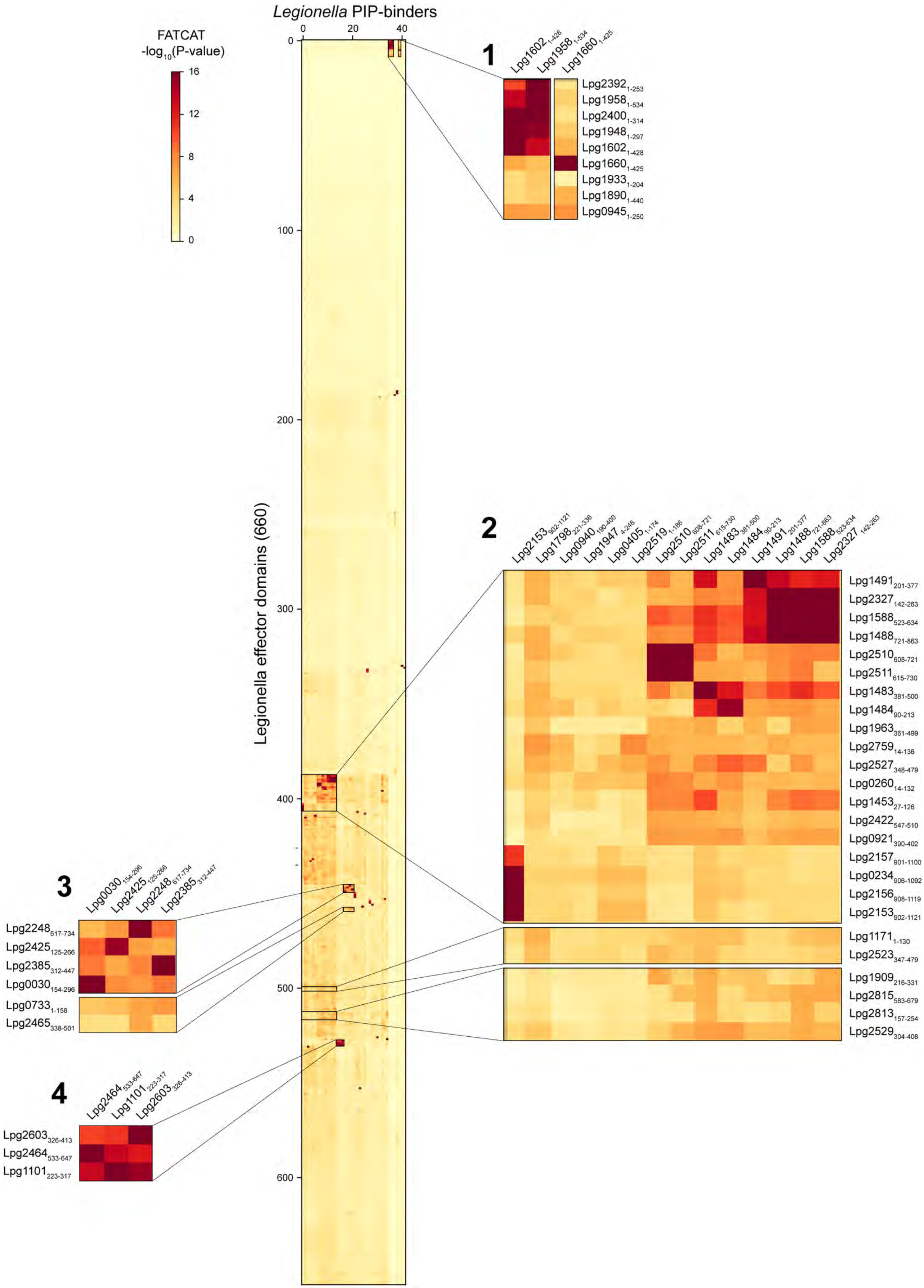

Legionella PIP-binders
FATCAT
-log10(P-value)
16
12
8
4
0
0
20
40
0
100
200
300
400
500
600
Legionella effector domains (660)
1
Lpg1602 1-428
Lpg1958 1-534
Lpg1660 1-425
Lpg2392 1-253
Lpg1958 1-534
Lpg2400 1-314
Lpg1948 1-297
Lpg1602 1-428
Lpg1660 1-425
Lpg1933 1-204
Lpg1890 1-440
Lpg0945 1-250
2
Lpg2153 902-1121
Lpg1798 221-336
Lpg0940 190-400
Lpg1947 4-248
Lpg0405 1-174
Lpg2519 1-186
Lpg2510 608-721
Lpg2511 615-730
Lpg1483 381-500
Lpg1484 90-213
Lpg1491 201-377
Lpg1488 721-863
Lpg1588 523-634
Lpg2327 142-263
Lpg1491 201-377
Lpg2327 142-263
Lpg1588 523-634
Lpg1488 721-863
Lpg2510 608-721
Lpg2511 615-730
Lpg1483 381-500
Lpg1484 90-213
Lpg1963 361-499
Lpg2759 14-136
Lpg2527 348-479
Lpg0260 14-132
Lpg1453 27-126
Lpg2422 547-510
Lpg0921 390-402
Lpg2157 901-1100
Lpg0234 906-1092
Lpg2156 908-1119
Lpg2153 902-1121
Lpg1171 1-130
Lpg2523 347-479
Lpg1909 216-331
Lpg2815 583-679
Lpg2813 157-254
Lpg2529 304-408
3
Lpg0030 154-296
Lpg2425 125-266
Lpg2248 617-734
Lpg2385 312-447
Lpg2248 617-734
Lpg2425 125-266
Lpg2385 312-447
Lpg0030 154-296
Lpg0733 1-158
Lpg2465 338-501
4
Lpg2464 533-647
Lpg1101 223-317
Lpg2603 326-413
Lpg2603 326-413
Lpg2464 533-647
Lpg1101 223-317

Fig. S6. Asymmetric similarity matrix of the -log$_{10}$(P-value) values from FATCAT analysis of *Legionella* PIP-binding effectors against *Legionella* effector domain database. A matrix was generated using the -log$_{10}$(P-value) from the comparison of PIP-binding effector domain structures (or full-length protein when the domain was unknown) to 660 *Legionella* effector domains by FATCAT. Axes are clustered by Ward's method and selected areas of interest are magnified. Area 1 contains the Leucine-rich repeat containing effectors. Area 2 contains PIP-binding effectors with helical binding regions including P4C (Lpg2510/SidC, Lpg2511/SdcA) and LED006 domain-containing effectors (Lpg1483/LegK1, Lpg1484/Ceg22, Lpg1491/Lem9, Lpg1488/Lgt3, Lpg1588/LegC6, and Lpg2327) as well as additional PIP-binding effectors identified in this study and candidate PIP-binding effectors containing structurally similar helical folds. Area 3 contains the three effectors possessing LED035 (Lpg0030/RavB, Lpg2425/MavH, and Lpg2248/LotA) as well as the newly identified PIP-binding effector Lpg2385. Additionally, Lpg0733/RavH and Lpg2465/SidD cluster near this group. Area 4 contains the three effectors with the P4M domain (Lpg2464/SidM, Lpg1101, and Lpg2603).

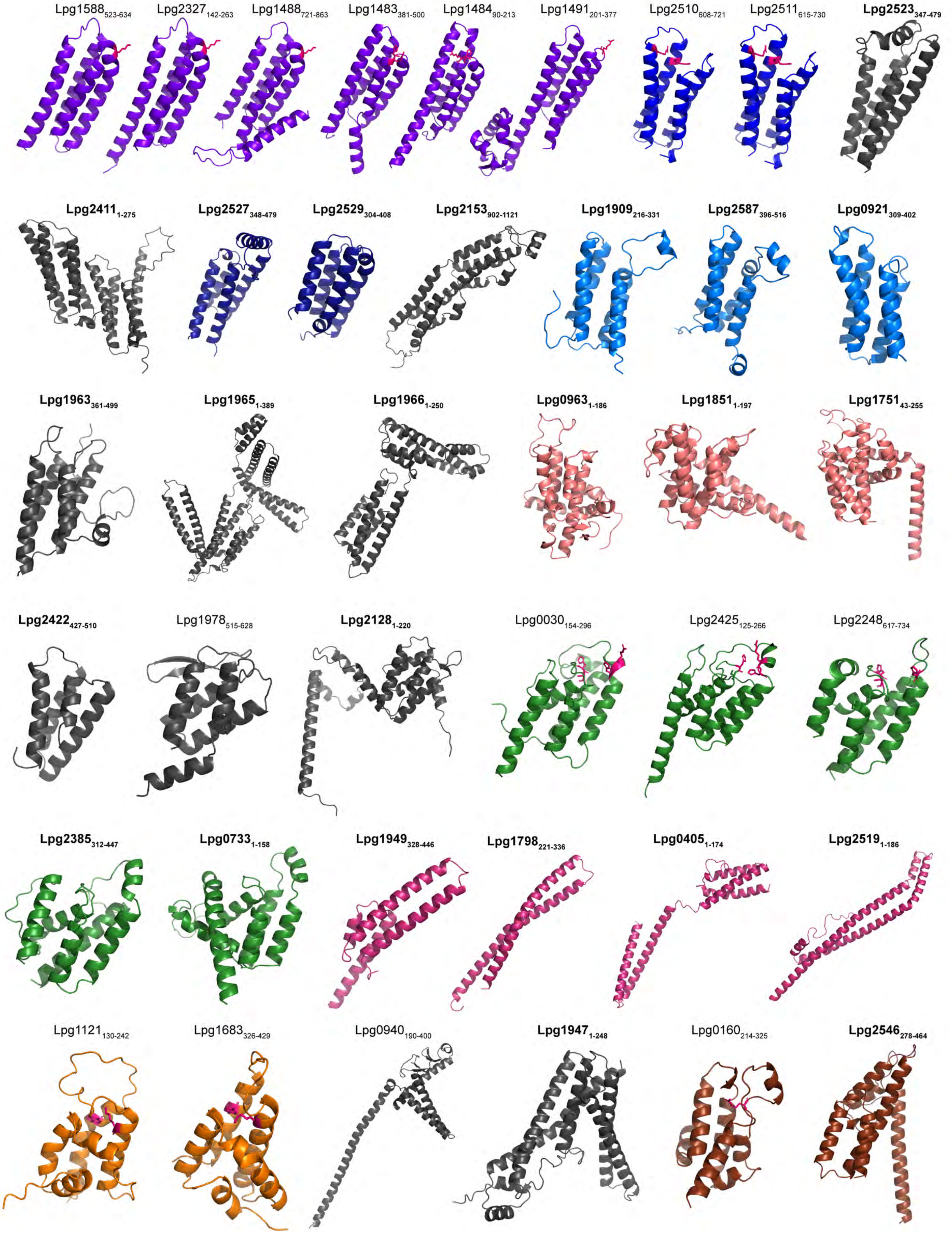
Lpg1588$_{523-634}$
Lpg2327$_{142-263}$
Lpg1488$_{721-863}$
Lpg1483$_{381-500}$
Lpg1484$_{90-213}$
Lpg1491$_{201-377}$
Lpg2510$_{608-721}$
Lpg2511$_{615-730}$
Lpg2523$_{347-479}$
Lpg2411$_{1-275}$
Lpg2527$_{348-479}$
Lpg2529$_{304-408}$
Lpg2153$_{902-1121}$
Lpg1909$_{216-331}$
Lpg2587$_{396-516}$
Lpg0921$_{309-402}$
Lpg1963$_{361-499}$
Lpg1965$_{1-389}$
Lpg1966$_{1-250}$
Lpg0963$_{1-186}$
Lpg1851$_{1-197}$
Lpg1751$_{43-255}$
Lpg2422$_{427-510}$
Lpg1978$_{515-628}$
Lpg2128$_{1-220}$
Lpg0030$_{154-296}$
Lpg2425$_{125-266}$
Lpg2248$_{617-734}$
Lpg2385$_{312-447}$
Lpg0733$_{1-158}$
Lpg1949$_{328-446}$
Lpg1798$_{221-336}$
Lpg0405$_{1-174}$
Lpg2519$_{1-186}$
Lpg1121$_{130-242}$
Lpg1683$_{326-429}$
Lpg0940$_{190-400}$
Lpg1947$_{1-248}$
Lpg0160$_{214-325}$
Lpg2546$_{278-464}$

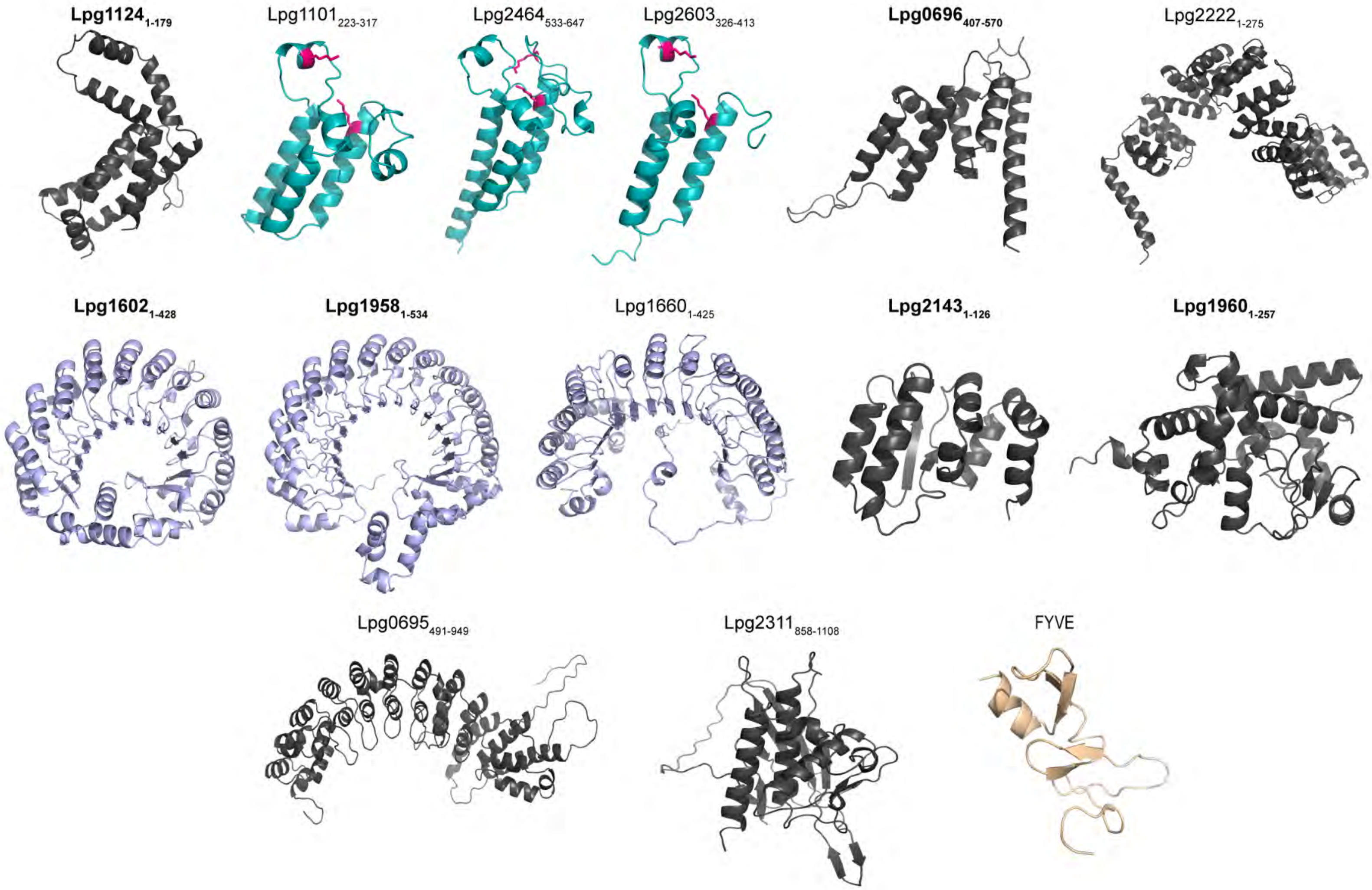

Fig. S7. AlphaFold structures of PIP-binding effectors or effector domains included in the dendrogram analysis. Structures are color-coded based on dendrogram groupings and branch color groupings in main figure 4. Labels in bold are PIP-binders discovered in this study. Known critical binding residues are depicted as pink sticks when applicable.

Table S1. PIP-binding effector proteins

PIP-binding effectors identified in this study:					
**Effector Protein**	**PIP target**	**PIP-binding region**	**Fold description**	**Function**	**Ref.**
Lpg0405	PI(3)P				
Lpg0696 Lem3	PI(3)P	407-570	7 α-helix	Dephosphocholinates Rab1, removing AnkX modification	1
Lpg0733 RavH	PI(3)P	1-158	5 α-helix bundle		
Lpg0921 MavT	PI(3)P	309-402	4 α-helix bundle		
Lpg0963	PI(3)P	1-186	Lpg1851 fold		2
Lpg1124	PI(3)P				
Lpg1602 LegL2	PI(3)P		Leucine-rich repeat		
Lpg1751	PI(3)P	43-255	Lpg1851 fold		2
Lpg1798 MavU	PI(3)P	221-336	2 α-helix anti-parallel		
Lpg1851 Lem14	PI(3)P	1-197	Small all α-helical protein	Synergistic with effector protein SidP, a PI3-phosphatase	3
Lpg1909 DenR	PI(3)P	216-331	4 α-helix bundle	Recruits nRas to LCV resulting in reduced MAPK signaling	4
Lpg1947 Lem16	PI(3)P				
Lpg1949 Lem17	PI(3)P	328-446	3 α-helix	Possesses acetyltransferase activity	5
Lpg1958 LegL5	PI(4)P PI(5)P; PS		Leucine-rich repeat		
Lpg1960 LirA	PI(3)P				
Lpg1963 PieA	PI(3)P	361-499	4 α-helix bundle	Localizes to LCV and disrupts lysosome morphology; on island of genomic plasticity	6
Lpg1965 PieC	PI(3)P	1-389		On island of genomic plasticity	6
Lpg1966 PieD	PI(3)P	1-250		On island of genomic plasticity	6

Lpg2128	PI(3)P				
Lpg2143	PI(3)P				
Lpg2153 SdeC	PI(3)P	902-1121	10 α-helix bundle	Phosphoribosyl ubiquitination of host proteins including Rtn4 resulting in ER rearrangement	[7-9]
Lpg2385	PI(3)P $PI(3,5)P_2$	312-447	5 α-helix bundle		
Lpg2411 Lem24	PI(3)P				
Lpg2422 Lem25	PI(3)P PS		4 α-helix bundle		
Lpg2519	PI(3)P PI(5)P		2 α-helix anti-parallel		
Lpg2523 Lem26	PI(3)P	347-479	4 α-helix bundle		
Lpg2527 LnaB	PI(3)P PI(5)P	348-479	4 α-helix bundle	Activates NF-κB; AMPylase of SidE generated PR-Ub, activity activated by actin binding	[10-12]
Lpg2529 Lem27	PI(3)P	304-408	4 α-helix bundle	Deubiquitinase; localizes to the LCV and regulated Rab10 ubiquitination levels	[13]
Lpg2546	PI(3)P PI(4)P	278-464	7 α-helix bundle		
Lpg2587	PI(3)P	391-516	4 α-helix bundle		

Previously known PIP-binding effectors:

Effector Protein	PIP target	PIP-binding region	Fold description	Function	Ref.
Lpg0030 RavB	PI(3)P	721-863 LED035	5 α-helix bundle		[14]
Lpg0160 RavD	PI(3)P	214-325	7 α-helix bundle	Deubiquitinase that removes ubiquitin from the LCV, prevents lysosomal fusion with LCV, and inhibits NF-κB signaling	[15,16]
Lpg0695 AnkX	PI(3)P; PI(4)P	491-949		Phosphocholinates Rab1 and 35; modulates endocytic traffic	[17-20]
Lpg0940 LidA	PI(3)P; PI(4)P	190-400		Facilitates Rab1 recruitment to the LCV and stabilizes active Rab1	[21-24]
Lpg1101 Lem4	PI(4)P	223-317 P4M	6 α-helix bundle	Phosphotyrosine phosphatase that localizes to lysosomes	[25,26]
Lpg1121	PI(3)P	13-242	5 α-helix bundle		[14]

Ceg19		LED027			
Lpg1483<br>LegK1	PI(3)P	381-500<br>LED006	4 α-helix bundle	Activates NF-κB pathway through phosphorylation of IκB alpha	[14,27]
Lpg1484<br>Ceg22	PI(3)P	90-213<br>LED006	4 α-helix bundle		[14]
Lpg1488<br>LegC5/Lgt3	PI(3)P	721-863<br>LED006	4 α-helix bundle	Glycosyltransferase of eEF1A, inhibits protein translation	[14,28,29]
Lpg1491<br>Lem9	PI(3)P	201-377<br>LED006	4 α-helix bundle		[14]
Lpg1588<br>LegC6	PI(3)P	523-634<br>LED006	4 α-helix bundle		[14]
Lpg1660<br>LegL3	PI(3)P; PS		Leucine-rich repeat		[30]
Lpg1683<br>RavZ	PI(3)P	326-429<br>LED027	5 α-helix bundle	Inhibits autophagy through the irreversible deconjugation of Atg8 on autophagosomes	[14,31,32]
Lpg1978<br>SetA	PI(3)P	615-728	4 α-helix bundle	Promiscuous glycosyltransferase activated by PI(3)P-binding; disrupts vesicular traffic	[33-35]
Lpg2222<br>LpnE	PI(3)P		ARM repeat	Interacts with OCLR1 on the LCV and assists with LCV biogenesis	[36]
Lpg2248<br>Lem21/LotA	PI(3)P	617-734<br>LED035	5 α-helix bundle	Localizes to the LCV where it cleaves ubiquitin chains through deubiquitinase activity	[14,37]
Lpg2311<br>RidL	PI(3)P	858-1108		Binds to the retromer complex and inhibits retrograde traffic	[38,39]
Lpg2327	PI(3)P	348-479<br>LED006	4 α-helix bundle		[14]
Lpg2425<br>MavH	PI(3)P	125-266<br>LED035	5 α-helix bundle	Interacts with actin capping protein to stimulate actin polymerization	[14,40]
Lpg2464<br>SidM	PI(4)P	533-647<br>P4M	6 α-helix bundle	Recruits and activates Rab1 through GEF activity at LCV, locks Rab1 in active state by AMPylation	[21,24,41-47]
Lpg2490<br>LepB	PI(3)P	1210-1294<br>LED006	4 α-helix bundle	Rab1 GAP; PI4-kinase that generates PI(4)P on the LCV	[14,47,48]
Lpg2510<br>SdcA	PI(4)P	608-721<br>P4C	4 α-helix bundle	Paralog of Lpg2511 (SidC)	[49-52]
Lpg2511<br>SidC	PI(4)P	615-730<br>P4C	4 α-helix bundle	Recruits ER-derived vesicles to the LCV; E3 ubiquitin ligase	[49-52]
Lpg2603	PI(4)P	326-413	6 α-helix bundle	Kinase activated by inositol hexaphosphate	[25,53]

Lem28		P4M			

Table S2. Strains and plasmids used in this study

Strains or plasmid	Relevant features	Source or Reference
***E. coli* strains**		
2T1	*F-mcrA Δ(mrr-hsdRMS-mcrBC) Φ80lacZΔM15 ΔlacX74 recA1 araΔ139 Δ(ara-leu)7697 galU galK rpsL (StrR) endA1 nupG fhuA::IS2*	ThermoFisher Scientific
GC5	*F− ϕ80lacZΔM15 Δ(lacZYA-argF)U169 recA1 endA1 hsdR17(rK− mK+) phoA supE44 thi-1 gyrA96 relA1λ−tonA*	ThermoFisher Scientific
BL21 (DE3)	*F−ompT hsdS*$_B$*(r*$_B$ *− m*$_B$ *−) gal dcm(DE3)*	ThermoFisher Scientific
BL21 (DE3)pLysS	*F−ompT hsdS*$_B$*(r*$_B$ *− m*$_B$ *−) gal dcm(DE3) pLysS (CamR)*	ThermoFisher Scientific
**Plasmids**		
***E. coli* expression vectors**		
pDONR221-effector library	Gateway entry vector with effectors; Kan^r	Machner, NIH
pDONR221-*lpg0696*$_{407-560}$	pDONR221 carrying *lpg0696*$_{407-560}$; Kan^r	This study
pDONR221-*lpg1798*$_{221-336}$	pDONR221 carrying *lpg1798*$_{221-336}$; Kan^r	This study
pDONR221-*lpg2153*$_{903-1121}$	pDONR221 carrying *lpg2153*$_{902-1121}$ *;* Kan^r	This study
pDONR221-*lpg2385*$_{289-447}$	pDONR221 carrying *lpg2385*$_{289-447}$ *;* Kan^r	This study
pDONR221-*lpg2546*$_{278-461}$	pDONR221 carrying *lpg2546*$_{278-464}$ *;* Kan^r	This study
pDONR221-*lpg2587*$_{391-516}$	pDONR221 carrying *lpg2587*$_{391-516}$ *;* Kan^r	This study
pDEST17-effector library	Encodes 6×His- effectors; Amp^r	Machner, NIH
pDEST15	Encodes GST; Amp^r	ThermoFisher Scientific
pDEST15-*lpg0405*	Encoding IPTG-inducible expression of Lpg0405 with N-terminal GST tag; Amp^r	This study
pDEST15-*lpg0696*	Encoding IPTG-inducible expression of Lpg0696 with N-terminal GST tag; Amp^r	This study
pDEST15-*lpg0696*$_{407-560}$	Encoding IPTG-inducible expression of Lpg0696$_{407-560}$ with N-terminal GST tag; Amp^r	This study
pDEST15-*lpg0733*	Encoding IPTG-inducible expression of Lpg0733 with N-terminal GST tag; Amp^r	This study
pDEST15-*lpg0921*	Encoding IPTG-inducible expression of Lpg0921 with N-terminal GST tag; Amp^r	This study
pDEST15-*lpg0963*	Encoding IPTG-inducible expression of Lpg0963 with N-terminal GST tag; Amp^r	This study
pDEST15-*lpg0969*	Encoding IPTG-inducible expression of Lpg0969 with N-terminal GST tag; Amp^r	This study
pDEST15-*lpg1124*	Encoding IPTG-inducible expression of Lpg1124 with N-terminal GST tag; Amp^r	This study
pDEST15-*lpg1148*	Encoding IPTG-inducible expression of Lpg1148 with N-terminal GST tag; Amp^r	This study
pDEST15-*lpg1602*	Encoding IPTG-inducible expression of Lpg1602 with N-terminal GST tag; Amp^r	This study
pDEST15-*lpg1689*	Encoding IPTG-inducible expression of Lpg1689 with N-terminal GST tag; Amp^r	This study
pDEST15-*lpg1751*	Encoding IPTG-inducible expression of Lpg1751 with N-terminal GST tag; Amp^r	This study
pDEST15-*lpg1798*	Encoding IPTG-inducible expression of Lpg1798 with N-terminal GST tag; Amp^r	This study

pDEST15-*lpg1798$_{221\text{-}336}$*	Encoding IPTG-inducible expression of Lpg1798$_{221\text{-}336}$ with N-terminal GST tag; Amp^r	This study
pDEST15-*lpg1851*	Encoding IPTG-inducible expression of Lpg1851 with N-terminal GST tag; Amp^r	This study
pDEST15-*lpg1888*	Encoding IPTG-inducible expression of Lpg1888 with N-terminal GST tag; Amp^r	This study
pDEST15-*lpg1890*	Encoding IPTG-inducible expression of Lpg1890 with N-terminal GST tag; Amp^r	This study
pDEST15-*lpg1909*	Encoding IPTG-inducible expression of Lpg1909 with N-terminal GST tag; Amp^r	This study
pDEST15-*lpg1947*	Encoding IPTG-inducible expression of Lpg1947 with N-terminal GST tag; Amp^r	This study
pDEST15-*lpg1949*	Encoding IPTG-inducible expression of Lpg1949 with N-terminal GST tag; Amp^r	This study
pDEST15-*lpg1958*	Encoding IPTG-inducible expression of Lpg1958 with N-terminal GST tag; Amp^r	This study
pDEST15-*lpg1960*	Encoding IPTG-inducible expression of Lpg1960 with N-terminal GST tag; Amp^r	This study
pDEST15-*lpg1963*	Encoding IPTG-inducible expression of Lpg1963 with N-terminal GST tag; Amp^r	This study
pDEST15-*lpg1965*	Encoding IPTG-inducible expression of Lpg1965 with N-terminal GST tag; Amp^r	This study
pDEST15-*lpg1966*	Encoding IPTG-inducible expression of Lpg1966 with N-terminal GST tag; Amp^r	This study
pDEST15-*lpg2048*	Encoding IPTG-inducible expression of Lpg2048 with N-terminal GST tag; Amp^r	This study
pDEST15-*lpg2128*	Encoding IPTG-inducible expression of Lpg2128 with N-terminal GST tag; Amp^r	This study
pDEST15-*lpg2143*	Encoding IPTG-inducible expression of Lpg2143 with N-terminal GST tag; Amp^r	This study
pDEST15-*lpg2153*	Encoding IPTG-inducible expression of Lpg2153 with N-terminal GST tag; Amp^r	This study
pDEST15-*lpg2153$_{902\text{-}1121}$*	Encoding IPTG-inducible expression of Lpg2153$_{902\text{-}1121}$ with N-terminal GST tag; Amp^r	This study
pDEST15-*lpg2385*	Encoding IPTG-inducible expression of Lpg2385 with N-terminal GST tag; Amp^r	This study
pDEST15-*lpg2385$_{289\text{-}447}$*	Encoding IPTG-inducible expression of Lpg2385$_{289\text{-}447}$ with N-terminal GST tag; Amp^r	This study
pDEST15-*lpg2411*	Encoding IPTG-inducible expression of Lpg2411 with N-terminal GST tag; Amp^r	This study
pDEST15-*lpg2422*	Encoding IPTG-inducible expression of Lpg2422 with N-terminal GST tag; Amp^r	This study
pDEST15-*lpg2519*	Encoding IPTG-inducible expression of Lpg2519 with N-terminal GST tag; Amp^r	This study
pDEST15-*lpg2523*	Encoding IPTG-inducible expression of Lpg2523 with N-terminal GST tag; Amp^r	This study
pDEST15-*lpg2527*	Encoding IPTG-inducible expression of Lpg2527 with N-terminal GST tag; Amp^r	This study
pDEST15-*lpg2529*	Encoding IPTG-inducible expression of Lpg2529 with N-terminal GST tag; Amp^r	This study
pDEST15-*lpg2546*	Encoding IPTG-inducible expression of Lpg2546 with N-terminal GST tag; Amp^r	This study
pDEST15-*lpg2546$_{278\text{-}464}$*	Encoding IPTG-inducible expression of Lpg2546$_{278\text{-}464}$ with N-terminal GST tag; Amp^r	This study

pDEST15-*lpg2587*	Encoding IPTG-inducible expression of Lpg2587 with N-terminal GST tag; Amp^r	This study
pDEST15-*lpg2587*$_{391-516}$	Encoding IPTG-inducible expression of Lpg2587$_{391-517}$ with N-terminal GST tag; Amp^r	This study
pDEST15-*lpg2745*	Encoding IPTG-inducible expression of Lpg2745 with N-terminal GST tag; Amp^r	This study
pGEX-6P-1-*lpg1963*$_{361-499}$	Encoding IPTG-inducible expression of Lpg1963$_{361-499}$ with N-terminal cleavable GST tag; Amp^r	GenScript
pGEX-6P-1-*cbu1677*	Encoding IPTG-inducible expression of CBU1677 with N-terminal cleavable GST tag; Amp^r	GenScript
pGEX-6P-1-*cbu0041*$_{1-200}$	Encoding IPTG-inducible expression of CBU0041$_{1-200}$ with N-terminal cleavable GST tag; Amp^r	GenScript
pGEX-6P-1-b*opA*$_{350-512}$	Encoding IPTG-inducible expression of BopA$_{350-512}$ with N-terminal cleavable GST tag; Amp^r	GenScript
**Mammalian expression vectors**		
pEGFP-2×FYVE	Encoding GFP-tagged HRS tandem FYVE domain	[54]
EGFP-2×P4M	Encoding GFP-tagged tandem P4M domain, Kan^r	[43]
362 pCS mCherry-DEST	Encodes mCherry; Cam^r, Amp^r	[55]
362 pCS Cherry-*lpg0056*	Encoding mCherry-tagged Lpg0056, Cam^r, Amp^r	This study
362 pCS Cherry-*lpg0130*	Encoding mCherry-tagged Lpg0130, Cam^r, Amp^r	This study
362 pCS Cherry-*lpg0191*	Encoding mCherry-tagged Lpg0191, Cam^r, Amp^r	This study
362 pCS Cherry-*lpg0301*	Encoding mCherry-tagged Lpg0301, Cam^r, Amp^r	This study
362 pCS Cherry-*lpg0375*	Encoding mCherry-tagged Lpg0375, Cam^r, Amp^r	This study
362 pCS Cherry-*lpg0390*	Encoding mCherry-tagged Lpg0390, Cam^r, Amp^r	This study
362 pCS Cherry-*lpg0405*	Encoding mCherry-tagged Lpg0405, Cam^r, Amp^r	This study
362 pCS Cherry-*lpg0696*	Encoding mCherry-tagged Lpg0696, Cam^r, Amp^r	This study
362 pCS Cherry-*lpg0733*	Encoding mCherry-tagged Lpg0733, Cam^r, Amp^r	This study
362 pCS Cherry-*lpg0926*	Encoding mCherry-tagged Lpg0926, Cam^r, Amp^r	This study
362 pCS Cherry-*lpg0944*	Encoding mCherry-tagged Lpg0944, Cam^r, Amp^r	This study
362 pCS Cherry-*lpg0967*	Encoding mCherry-tagged Lpg0967, Cam^r, Amp^r	This study
362 pCS Cherry-*lpg0969*	Encoding mCherry-tagged Lpg0969, Cam^r, Amp^r	This study
362 pCS Cherry-*lpg1106*	Encoding mCherry-tagged Lpg1106, Cam^r, Amp^r	This study
362 pCS Cherry-*lpg1109*	Encoding mCherry-tagged Lpg1109, Cam^r, Amp^r	This study
362 pCS Cherry-*lpg1111*	Encoding mCherry-tagged Lpg1111, Cam^r, Amp^r	This study
362 pCS Cherry-*lpg1120*	Encoding mCherry-tagged Lpg1120, Cam^r, Amp^r	This study
362 pCS Cherry-*lpg1124*	Encoding mCherry-tagged Lpg1124, Cam^r, Amp^r	This study
362 pCS Cherry-*lpg1129*	Encoding mCherry-tagged Lpg1129, Cam^r, Amp^r	This study
362 pCS Cherry-*lpg1137*	Encoding mCherry-tagged Lpg1137, Cam^r, Amp^r	This study
362 pCS Cherry-*lpg1145*	Encoding mCherry-tagged Lpg1145, Cam^r, Amp^r	This study
362 pCS Cherry-*lpg1148*	Encoding mCherry-tagged Lpg1148, Cam^r, Amp^r	This study
362 pCS Cherry-*lpg1154*	Encoding mCherry-tagged Lpg1154, Cam^r, Amp^r	This study
362 pCS Cherry-*lpg1158*	Encoding mCherry-tagged Lpg1158, Cam^r, Amp^r	This study
362 pCS Cherry-*lpg1166*	Encoding mCherry-tagged Lpg1166, Cam^r, Amp^r	This study
362 pCS Cherry-*lpg1183*	Encoding mCherry-tagged Lpg1183, Cam^r, Amp^r	This study
362 pCS Cherry-*lpg1227*	Encoding mCherry-tagged Lpg1227, Cam^r, Amp^r	This study
362 pCS Cherry-*lpg1290*	Encoding mCherry-tagged Lpg1290, Cam^r, Amp^r	This study
362 pCS Cherry-*lpg1355*	Encoding mCherry-tagged Lpg1355, Cam^r, Amp^r	This study
362 pCS Cherry-*lpg1368*	Encoding mCherry-tagged Lpg1368, Cam^r, Amp^r	This study

362 pCS Cherry-*lpg1408*	Encoding mCherry-tagged Lpg1408, Cam^r, Amp^r	This study
362 pCS Cherry-*lpg1426*	Encoding mCherry-tagged Lpg1426, Cam^r, Amp^r	This study
362 pCS Cherry-*lpg1496*	Encoding mCherry-tagged Lpg1496, Cam^r, Amp^r	This study
362 pCS Cherry-*lpg1602*	Encoding mCherry-tagged Lpg1602, Cam^r, Amp^r	This study
362 pCS Cherry-*lpg1625*	Encoding mCherry-tagged Lpg1625, Cam^r, Amp^r	This study
362 pCS Cherry-*lpg1666*	Encoding mCherry-tagged Lpg1666, Cam^r, Amp^r	This study
362 pCS Cherry-*lpg1687*	Encoding mCherry-tagged Lpg1687, Cam^r, Amp^r	This study
362 pCS Cherry-*lpg1689*	Encoding mCherry-tagged Lpg1689, Cam^r, Amp^r	This study
362 pCS Cherry-*lpg1692*	Encoding mCherry-tagged Lpg1692, Cam^r, Amp^r	This study
362 pCS Cherry-*lpg1701*	Encoding mCherry-tagged Lpg1701, Cam^r, Amp^r	This study
362 pCS Cherry-*lpg1717*	Encoding mCherry-tagged Lpg1717, Cam^r, Amp^r	This study
362 pCS Cherry-*lpg1751*	Encoding mCherry-tagged Lpg1751, Cam^r, Amp^r	This study
362 pCS Cherry-*lpg1798*	Encoding mCherry-tagged Lpg1798, Cam^r, Amp^r	This study
362 pCS Cherry-*lpg1851*	Encoding mCherry-tagged Lpg1851, Cam^r, Amp^r	This study
362 pCS Cherry-*lpg1884*	Encoding mCherry-tagged Lpg1884, Cam^r, Amp^r	This study
362 pCS Cherry-*lpg1888*	Encoding mCherry-tagged Lpg1888, Cam^r, Amp^r	This study
362 pCS Cherry-*lpg1890*	Encoding mCherry-tagged Lpg1890, Cam^r, Amp^r	This study
362 pCS Cherry-*lpg1895*	Encoding mCherry-tagged Lpg1895, Cam^r, Amp^r	This study
362 pCS Cherry-*lpg1947*	Encoding mCherry-tagged Lpg1947, Cam^r, Amp^r	This study
362 pCS Cherry-*lpg1949*	Encoding mCherry-tagged Lpg1949, Cam^r, Amp^r	This study
362 pCS Cherry-*lpg1953*	Encoding mCherry-tagged Lpg1953, Cam^r, Amp^r	This study
362 pCS Cherry-*lpg1958*	Encoding mCherry-tagged Lpg1958, Cam^r, Amp^r	This study
362 pCS Cherry-*lpg1960*	Encoding mCherry-tagged Lpg1960, Cam^r, Amp^r	This study
362 pCS Cherry-*lpg1961*	Encoding mCherry-tagged Lpg1961, Cam^r, Amp^r	This study
362 pCS Cherry-*lpg1963*	Encoding mCherry-tagged Lpg1963, Cam^r, Amp^r	This study
362 pCS Cherry-*lpg1964*	Encoding mCherry-tagged Lpg1964, Cam^r, Amp^r	This study
362 pCS Cherry-*lpg1965*	Encoding mCherry-tagged Lpg1965, Cam^r, Amp^r	This study
362 pCS Cherry-*lpg1966*	Encoding mCherry-tagged Lpg1966, Cam^r, Amp^r	This study
362 pCS Cherry-*lpg1972*	Encoding mCherry-tagged Lpg1972, Cam^r, Amp^r	This study
362 pCS Cherry-*lpg1982*	Encoding mCherry-tagged Lpg1982, Cam^r, Amp^r	This study
362 pCS Cherry-*lpg2048*	Encoding mCherry-tagged Lpg2048, Cam^r, Amp^r	This study
362 pCS Cherry-*lpg2128*	Encoding mCherry-tagged Lpg2128, Cam^r, Amp^r	This study
362 pCS Cherry-*lpg2143*	Encoding mCherry-tagged Lpg2143, Cam^r, Amp^r	This study
362 pCS Cherry-*lpg2148*	Encoding mCherry-tagged Lpg2148, Cam^r, Amp^r	This study
362 pCS Cherry-*lpg2153*	Encoding mCherry-tagged Lpg2153, Cam^r, Amp^r	This study
362 pCS Cherry-*lpg2155*	Encoding mCherry-tagged Lpg2155, Cam^r, Amp^r	This study
362 pCS Cherry-*lpg2166*	Encoding mCherry-tagged Lpg2166, Cam^r, Amp^r	This study
362 pCS Cherry-*lpg2199*	Encoding mCherry-tagged Lpg2199, Cam^r, Amp^r	This study
362 pCS Cherry-*lpg2244*	Encoding mCherry-tagged Lpg2244, Cam^r, Amp^r	This study
362 pCS Cherry-*lpg2324*	Encoding mCherry-tagged Lpg2324, Cam^r, Amp^r	This study
362 pCS Cherry-*lpg2328*	Encoding mCherry-tagged Lpg2328, Cam^r, Amp^r	This study
362 pCS Cherry-*lpg2351*	Encoding mCherry-tagged Lpg2351, Cam^r, Amp^r	This study
362 pCS Cherry-*lpg2385*	Encoding mCherry-tagged Lpg2385, Cam^r, Amp^r	This study
362 pCS Cherry-*lpg2406*	Encoding mCherry-tagged Lpg2406, Cam^r, Amp^r	This study
362 pCS Cherry-*lpg2411*	Encoding mCherry-tagged Lpg2411, Cam^r, Amp^r	This study
362 pCS Cherry-*lpg2422*	Encoding mCherry-tagged Lpg2422, Cam^r, Amp^r	This study
362 pCS Cherry-*lpg2452*	Encoding mCherry-tagged Lpg2452, Cam^r, Amp^r	This study
362 pCS Cherry-*lpg2456*	Encoding mCherry-tagged Lpg2456, Cam^r, Amp^r	This study
362 pCS Cherry-*lpg2498*	Encoding mCherry-tagged Lpg2498, Cam^r, Amp^r	This study
362 pCS Cherry-*lpg2519*	Encoding mCherry-tagged Lpg2519, Cam^r, Amp^r	This study
362 pCS Cherry-*lpg2529*	Encoding mCherry-tagged Lpg2529, Cam^r, Amp^r	This study
362 pCS Cherry-*lpg2539*	Encoding mCherry-tagged Lpg2539, Cam^r, Amp^r	This study
362 pCS Cherry-*lpg2546*	Encoding mCherry-tagged Lpg2546, Cam^r, Amp^r	This study

362 pCS Cherry-*lpg2587*	Encoding mCherry-tagged Lpg2587, Cam^r, Amp^r	This study
362 pCS Cherry-*lpg2745*	Encoding mCherry-tagged Lpg2745, Cam^r, Amp^r	This study
362 pCS Cherry-*lpg2759*	Encoding mCherry-tagged Lpg2759, Cam^r, Amp^r	This study
362 pCS Cherry-*lpg2793*	Encoding mCherry-tagged Lpg2793, Cam^r, Amp^r	This study
362 pCS Cherry-*lpg2831*	Encoding mCherry-tagged Lpg2831, Cam^r, Amp^r	This study
362 pCS Cherry-*lpg2871*	Encoding mCherry-tagged Lpg2871, Cam^r, Amp^r	This study
362 pCS Cherry-*lpg2911*	Encoding mCherry-tagged Lpg2911, Cam^r, Amp^r	This study

Table S3. Oligonucleotides used in this study.

**Name**	**Sequence 5' to 3' direction**
Fwd-attB1-*lpg0696*$_{407\text{-}570}$	GGGGACAAGTTTGTACAAAAAAGCAGGCTTATTAGATGAGCTCATACG
Rev-attB2-*lpg0696*$_{407\text{-}570}$	GGGGACCACTTTGTACAAGAAAGCTGGGTTCTATTTCAATTCATTTTTATTTTCTATTTC
Fwd-attB1-*lpg1798*$_{221\text{-}336}$	GGGGACAAGTTTGTACAAAAAAGCAGGCTTAGAAAGGCATGCCGATAAATT
Rev-attB2-*lpg1798*$_{221\text{-}336}$	GGGGACCACTTTGTACAAGAAAGCTGGGTTTTAAGAGCGCGATAT
Fwd-attB1-*lpg1963*$_{361\text{-}499}$	GGGGACAAGTTTGTACAAAAAAGCAGGCTTAAAAGACCAAAAAGAGTTTTCTAAATT
Rev-attB2-*lpg1963*$_{361\text{-}499}$	GGGGACCACTTTGTACAAGAAAGCTGGGTTTTATCTCTTTTGAACCTG
Fwd-attB1-*lpg2153*$_{902\text{-}1121}$	GGGGACAAGTTTGTACAAAAAAGCAGGCTTACGACATGAAAGCGG
Rev-attB2-*lpg2153*$_{902\text{-}1121}$	GGGGACCACTTTGTACAAGAAAGCTGGGTTTTAAATTGGAGTAGTGGT
Fwd-attB1-*lpg2385*$_{289\text{-}447}$	GGGGACAAGTTTGTACAAAAAAGCAGGCTTATCAGATGAAAAAATTGACGTAGT
Rev-attB2-*lpg2385*$_{289\text{-}447}$	GGGGACCACTTTGTACAAGAAAGCTGGGTTTTACGTTTTTGCTAC
Fwd-attB1-*lpg2546*$_{278\text{-}464}$	GGGGACAAGTTTGTACAAAAAAGCAGGCTTACGATTTGGAAAAGATGA
Rev-attB2-*lpg2546*$_{278\text{-}464}$	GGGGACCACTTTGTACAAGAAAGCTGGGTTTTAAGATGAATGCTC
Fwd-attB1-*lpg2587*$_{391\text{-}516}$	GGGGACAAGTTTGTACAAAAAAGCAGGCTTATCCATGGTCAAAACCGATAA
Rev-attB2-*lpg2587*$_{391\text{-}516}$	GGGGACCACTTTGTACAAGAAAGCTGGGTTTTAGTCTGTTGTTTTTGCCAG

SI References

1 Tan, Y., Arnold, R. J. & Luo, Z. Q. Legionella pneumophila regulates the small GTPase Rab1 activity by reversible phosphorylcholination. *Proc Natl Acad Sci U S A* **108**, 21212–21217 (2011). https://doi.org/10.1073/pnas.1114023109

2 Patel, D. T. *et al.* Global atlas of predicted functional domains in Legionella pneumophila Dot/Icm translocated effectors. *Mol Syst Biol* **21**, 59–89 (2025). https://doi.org/10.1038/s44320-024-00076-z

3 Urbanus, M. L. *et al.* Diverse mechanisms of metaeffector activity in an intracellular bacterial pathogen, Legionella pneumophila. *Mol Syst Biol* **12**, 893 (2016). https://doi.org/10.15252/msb.20167381

4 Lehman, S. S. *et al.* The Legionella pneumophila effector DenR hijacks the host NRas proto-oncoprotein to downregulate MAPK signaling. *Cell Rep* **43**, 114033 (2024). https://doi.org/10.1016/j.celrep.2024.114033

5 Hermanns, T. *et al.* An evolutionary approach to systematic discovery of novel deubiquitinases, applied to Legionella. *Life Sci Alliance* **3** (2020). https://doi.org/10.26508/lsa.202000838

6 Ninio, S., Celli, J. & Roy, C. R. A Legionella pneumophila effector protein encoded in a region of genomic plasticity binds to Dot/Icm-modified vacuoles. *PLoS Pathog* **5**, e1000278 (2009). https://doi.org/10.1371/journal.ppat.1000278

7 Kotewicz, K. M. *et al.* A Single Legionella Effector Catalyzes a Multistep Ubiquitination Pathway to Rearrange Tubular Endoplasmic Reticulum for Replication. *Cell Host Microbe* **21**, 169–181 (2017). https://doi.org/10.1016/j.chom.2016.12.007

8 Qiu, J. *et al.* Ubiquitination independent of E1 and E2 enzymes by bacterial effectors. *Nature* **533**, 120–124 (2016). https://doi.org/10.1038/nature17657

9 Bhogaraju, S. *et al.* Phosphoribosylation of Ubiquitin Promotes Serine Ubiquitination and Impairs Conventional Ubiquitination. *Cell* **167**, 1636–1649 e1613 (2016). https://doi.org/10.1016/j.cell.2016.11.019

10 Losick, V. P., Haenssler, E., Moy, M. Y. & Isberg, R. R. LnaB: a Legionella pneumophila activator of NF-kappaB. *Cell Microbiol* **12**, 1083–1097 (2010). https://doi.org/10.1111/j.1462-5822.2010.01452.x

11 Wang, T. *et al.* Legionella effector LnaB is a phosphoryl-AMPylase that impairs phosphosignalling. *Nature* **631**, 393–401 (2024). https://doi.org/10.1038/s41586-024-07573-z

12 Fu, J. *et al.* Legionella maintains host cell ubiquitin homeostasis by effectors with unique catalytic mechanisms. *Nat Commun* **15**, 5953 (2024). https://doi.org/10.1038/s41467-024-50311-2

13 Liu, S. *et al.* Interplay between bacterial deubiquitinase and ubiquitin E3 ligase regulates ubiquitin dynamics on Legionella phagosomes. *Elife* **9** (2020). https://doi.org/10.7554/eLife.58114

14 Nachmias, N., Zusman, T. & Segal, G. Study of Legionella Effector Domains Revealed Novel and Prevalent Phosphatidylinositol 3-Phosphate Binding Domains. *Infect Immun* **87** (2019). https://doi.org/10.1128/iai.00153-19

15 Pike, C. M., Boyer-Andersen, R., Kinch, L. N., Caplan, J. L. & Neunuebel, M. R. The Legionella effector RavD binds phosphatidylinositol-3-phosphate and helps suppress endolysosomal maturation of the Legionella-containing vacuole. *J Biol Chem* **294**, 6405–6415 (2019). https://doi.org/10.1074/jbc.RA118.007086

16 Wan, M. *et al.* A bacterial effector deubiquitinase specifically hydrolyses linear ubiquitin chains to inhibit host inflammatory signalling. *Nat Microbiol* **4**, 1282–1293 (2019). https://doi.org/10.1038/s41564-019-0454-1

17 Allgood, S. C. *et al.* Legionella Effector AnkX Disrupts Host Cell Endocytic Recycling in a Phosphocholination-Dependent Manner. *Front Cell Infect Microbiol* **7**, 397 (2017). https://doi.org/10.3389/fcimb.2017.00397

18 Mukherjee, S. *et al.* Modulation of Rab GTPase function by a protein phosphocholine transferase. *Nature* **477**, 103–106 (2011). https://doi.org/10.1038/nature10335

19 Goody, P. R. *et al.* Reversible phosphocholination of Rab proteins by Legionella pneumophila effector proteins. *Embo j* **31**, 1774–1784 (2012). https://doi.org/10.1038/emboj.2012.16

20 Campanacci, V., Mukherjee, S., Roy, C. R. & Cherfils, J. Structure of the Legionella effector AnkX reveals the mechanism of phosphocholine transfer by the FIC domain. *Embo j* **32**, 1469–1477 (2013). https://doi.org/10.1038/emboj.2013.82

21 Brombacher, E. *et al.* Rab1 guanine nucleotide exchange factor SidM is a major phosphatidylinositol 4-phosphate-binding effector protein of Legionella pneumophila. *J Biol Chem* **284**, 4846–4856 (2009). https://doi.org/10.1074/jbc.M807505200

22 Neunuebel, M. R., Mohammadi, S., Jarnik, M. & Machner, M. P. Legionella pneumophila LidA affects nucleotide binding and activity of the host GTPase Rab1. *J Bacteriol* **194**, 1389–1400 (2012). https://doi.org/10.1128/jb.06306-11

23 Schoebel, S., Cichy, A. L., Goody, R. S. & Itzen, A. Protein LidA from Legionella is a Rab GTPase supereffector. *Proc Natl Acad Sci U S A* **108**, 17945–17950 (2011). https://doi.org/10.1073/pnas.1113133108

24 Machner, M. P. & Isberg, R. R. Targeting of host Rab GTPase function by the intravacuolar pathogen Legionella pneumophila. *Dev Cell* **11**, 47–56 (2006). https://doi.org/10.1016/j.devcel.2006.05.013

25 Hubber, A. *et al.* The machinery at endoplasmic reticulum-plasma membrane contact sites contributes to spatial regulation of multiple Legionella effector proteins. *PLoS Pathog* **10**, e1004222 (2014). https://doi.org/10.1371/journal.ppat.1004222

26 Beyrakhova, K., Li, L., Xu, C., Gagarinova, A. & Cygler, M. Legionella pneumophila effector Lem4 is a membrane-associated protein tyrosine phosphatase. *J Biol Chem* **293**, 13044–13058 (2018). https://doi.org/10.1074/jbc.RA118.003845

27 Ge, J. *et al.* A Legionella type IV effector activates the NF-kappaB pathway by phosphorylating the IkappaB family of inhibitors. *Proc Natl Acad Sci U S A* **106**, 13725–13730 (2009). https://doi.org/10.1073/pnas.0907200106

28 Shen, X. *et al.* Targeting eEF1A by a Legionella pneumophila effector leads to inhibition of protein synthesis and induction of host stress response. *Cell Microbiol* **11**, 911–926 (2009). https://doi.org/10.1111/j.1462-5822.2009.01301.x

29 Belyi, Y., Tabakova, I., Stahl, M. & Aktories, K. Lgt: a family of cytotoxic glucosyltransferases produced by Legionella pneumophila. *J Bacteriol* **190**, 3026–3035 (2008). https://doi.org/10.1128/JB.01798-07

30 Weigele, B. A., Orchard, R. C., Jimenez, A., Cox, G. W. & Alto, N. M. A systematic exploration of the interactions between bacterial effector proteins and host cell membranes. *Nat Commun* **8**, 532 (2017). https://doi.org/10.1038/s41467-017-00700-7

31 Horenkamp, F. A. *et al.* The Legionella Anti-autophagy Effector RavZ Targets the Autophagosome via PI3P- and Curvature-Sensing Motifs. *Dev Cell* **34**, 569–576 (2015). https://doi.org/10.1016/j.devcel.2015.08.010

32 Choy, A. *et al.* The Legionella effector RavZ inhibits host autophagy through irreversible Atg8 deconjugation. *Science* **338**, 1072–1076 (2012). https://doi.org/10.1126/science.1227026

33 Jank, T. *et al.* Domain organization of Legionella effector SetA. *Cell Microbiol* **14**, 852–868 (2012). https://doi.org/10.1111/j.1462-5822.2012.01761.x

34 Levanova, N. *et al.* Characterization of the glucosyltransferase activity of Legionella pneumophila effector SetA. *Naunyn Schmiedebergs Arch Pharmacol* **392**, 69–79 (2019). https://doi.org/10.1007/s00210-018-1562-9

35 Beck, W. H. J. *et al.* Glucosylation by the Legionella Effector SetA Promotes the Nuclear Localization of the Transcription Factor TFEB. *iScience* **23**, 101300 (2020). https://doi.org/10.1016/j.isci.2020.101300

36 Weber, S. S., Ragaz, C. & Hilbi, H. The inositol polyphosphate 5-phosphatase OCRL1 restricts intracellular growth of Legionella, localizes to the replicative vacuole and binds to the bacterial effector LpnE. *Cell Microbiol* **11**, 442–460 (2009). https://doi.org/10.1111/j.1462-5822.2008.01266.x

37 Kubori, T., Kitao, T., Ando, H. & Nagai, H. LotA, a Legionella deubiquitinase, has dual catalytic activity and contributes to intracellular growth. *Cell Microbiol* **20**, e12840 (2018). https://doi.org/10.1111/cmi.12840

38 Finsel, I. *et al.* The Legionella effector RidL inhibits retrograde trafficking to promote intracellular replication. *Cell Host Microbe* **14**, 38–50 (2013). https://doi.org/10.1016/j.chom.2013.06.001

39 Bärlocher, K. *et al.* Structural insights into Legionella RidL-Vps29 retromer subunit interaction reveal displacement of the regulator TBC1D5. *Nat Commun* **8**, 1543 (2017). https://doi.org/10.1038/s41467-017-01512-5

40 Zhang, Q., Wan, M., Kudryashova, E., Kudryashov, D. S. & Mao, Y. Membrane-dependent actin polymerization mediated by the Legionella pneumophila effector protein MavH. *PLoS Pathog* **19**, e1011512 (2023). https://doi.org/10.1371/journal.ppat.1011512

41 Machner, M. P. & Isberg, R. R. A bifunctional bacterial protein links GDI displacement to Rab1 activation. *Science* **318**, 974–977 (2007). https://doi.org/10.1126/science.1149121

42 Del Campo, C. M. *et al.* Structural basis for PI(4)P-specific membrane recruitment of the Legionella pneumophila effector DrrA/SidM. *Structure* **22**, 397–408 (2014). https://doi.org/10.1016/j.str.2013.12.018

43 Hammond, G. R., Machner, M. P. & Balla, T. A novel probe for phosphatidylinositol 4-phosphate reveals multiple pools beyond the Golgi. *J Cell Biol* **205**, 113–126 (2014). https://doi.org/10.1083/jcb.201312072

44 Arasaki, K., Toomre, D. K. & Roy, C. R. The Legionella pneumophila effector DrrA is sufficient to stimulate SNARE-dependent membrane fusion. *Cell Host Microbe* **11**, 46–57 (2012). https://doi.org/10.1016/j.chom.2011.11.009

45 Murata, T. *et al.* The Legionella pneumophila effector protein DrrA is a Rab1 guanine nucleotide-exchange factor. *Nat Cell Biol* **8**, 971–977 (2006). https://doi.org/10.1038/ncb1463

46 Schoebel, S., Blankenfeldt, W., Goody, R. S. & Itzen, A. High-affinity binding of phosphatidylinositol 4-phosphate by Legionella pneumophila DrrA. *EMBO Rep* **11**, 598–604 (2010). https://doi.org/10.1038/embor.2010.97

47 Ingmundson, A., Delprato, A., Lambright, D. G. & Roy, C. R. Legionella pneumophila proteins that regulate Rab1 membrane cycling. *Nature* **450**, 365–369 (2007). https://doi.org/10.1038/nature06336

48 Dong, N. *et al.* Modulation of membrane phosphoinositide dynamics by the phosphatidylinositide 4-kinase activity of the Legionella LepB effector. *Nat Microbiol* **2**, 16236 (2016). https://doi.org/10.1038/nmicrobiol.2016.236

49 Luo, X. *et al.* Structure of the Legionella Virulence Factor, SidC Reveals a Unique PI(4)P-Specific Binding Domain Essential for Its Targeting to the Bacterial Phagosome. *PLoS Pathog* **11**, e1004965 (2015). https://doi.org/10.1371/journal.ppat.1004965

50 Ragaz, C. *et al.* The Legionella pneumophila phosphatidylinositol-4 phosphate-binding type IV substrate SidC recruits endoplasmic reticulum vesicles to a replication-permissive vacuole. *Cell Microbiol* **10**, 2416–2433 (2008). https://doi.org/10.1111/j.1462-5822.2008.01219.x

51 Hsu, F. *et al.* The Legionella effector SidC defines a unique family of ubiquitin ligases important for bacterial phagosomal remodeling. *Proc Natl Acad Sci U S A* **111**, 10538–10543 (2014). https://doi.org/10.1073/pnas.1402605111

52 Wasilko, D. J., Huang, Q. & Mao, Y. Insights into the ubiquitin transfer cascade catalyzed by the Legionella effector SidC. *Elife* **7** (2018). https://doi.org/10.7554/eLife.36154

53 Sreelatha, A. *et al.* A Legionella effector kinase is activated by host inositol hexakisphosphate. *J Biol Chem* **295**, 6214–6224 (2020). https://doi.org/10.1074/jbc.RA120.013067

54 Gillooly, D. J. *et al.* Localization of phosphatidylinositol 3-phosphate in yeast and mammalian cells. *EMBO J* **19**, 4577–4588 (2000). https://doi.org/10.1093/emboj/19.17.4577

55 Villefranc, J. A., Amigo, J. & Lawson, N. D. Gateway compatible vectors for analysis of gene function in the zebrafish. *Dev Dyn* **236**, 3077–3087 (2007). https://doi.org/10.1002/dvdy.21354
